# Levelized cost-based learning analysis of utility-scale wind and solar in the United States

**DOI:** 10.1016/j.isci.2022.104378

**Published:** 2022-05-09

**Authors:** Mark Bolinger, Ryan Wiser, Eric O'Shaughnessy

**Affiliations:** 1 Lawrence Berkeley National Laboratory, Berkeley, CA 94720, USA

**Keywords:** Energy policy, Energy systems, Applied sciences

## Abstract

Learning curves play a central role in power sector planning. We improve upon past learning curves for utility-scale wind and solar through a combination of approaches. First, we generate plant-level estimates of the levelized cost of energy (LCOE) in the United States, and then use LCOE, rather than capital costs, as the dependent variable. Second, we normalize LCOE to control for exogenous influences unrelated to learning. Third, we use segmented regression to identify change points in LCOE learning. We find full-period LCOE-based learning rates of 15% for wind and 24% for solar, and conclude that (normalized) LCOE-based learning provides a more complete view of technology advancement than afforded by much of the existing literature—particularly that which focuses solely on capital cost learning. Models that do not account for endogenous LCOE-based learning, or that focus narrowly on capital cost learning, may underestimate future LCOE reductions.

## Highlights

• We estimate learning for wind and solar in the U.S. based on levelized costs
• We control for exogenous influences and use a segmented regression model
• We find levelized cost learning rates of 15% for wind and 24% for solar
• Models that focus on initial rather than levelized cost may underestimate learning

## Graphical abstract

**Figure:**
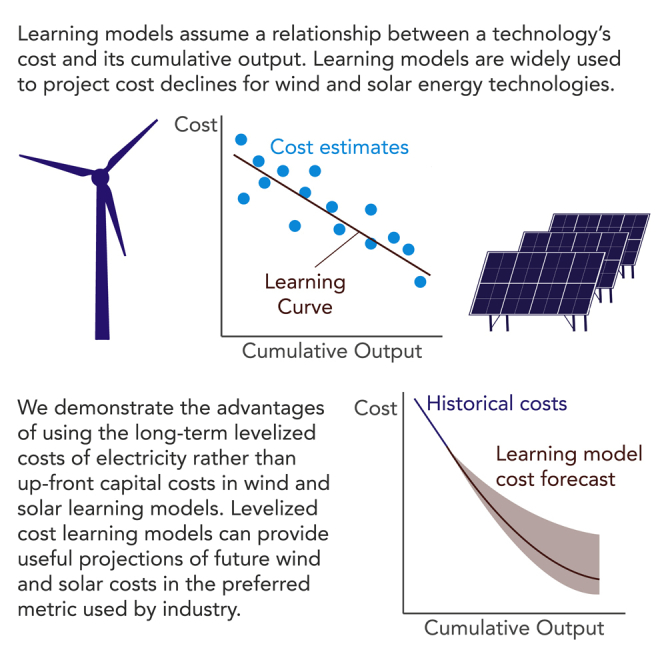

## Introduction

Mitigating the adverse consequences of climate change will require a transformation of the supply and use of energy (IPCC, 2018). A growing body of research suggests that wind and solar are likely to serve as central pillars to that transformation (Williams et al., 2021; National Academies, 2021; Creutzig et al., 2017; Luderer et al., 2017; Pietzcker et al., 2017; IEA, 2020). Others point out that various social, institutional, and power-system integration challenges will need to be overcome in order to achieve the envisioned growth rates for wind and solar (Cherp et al., 2021). Underlying the more optimistic expectations are past wind and solar cost reductions (Wiser et al., 2021a; Bolinger et al., 2021; LAZARD, 2020; Beiter et al., 2021; Apostoleris et al., 2018; IRENA, 2021) and forecasts that technological advancement will continue to drive costs lower in the decades ahead (Veers et al., 2019; Haegel et al., 2019; Green, 2019; Wiser et al., 2021b). Yet there are concerns that, among their other limitations (Cherp et al., 2021), energy planning models have not kept pace with the steep recent declines in wind and solar costs (Xiao et al., 2021; Krey et al., 2019), in part because such models do not always appropriately account for induced deployment-oriented learning (Grubb et al., 2021a). This paper focuses exclusively on understanding past and projecting future wind and solar costs, though we recognize that a variety of other critical factors also impact deployment volumes.

Analysts have employed a variety of methods to understand past wind and solar cost trends and to project future trends. Expert elicitation (Wiser et al., 2021b; Verdolini et al., 2018; Bosetti et al., 2012) and engineering assessments (Jones-Albertus et al., 2016; Bolinger et al., 2020a, 2020b) are regularly used, but learning (or experience) curves are the most common approach (Junginger and Louwen, 2020). Learning-by-doing (or simply learning) is a broadly accepted concept for explaining relationships between technology cost reductions and cumulative output. It posits that costs decline as a function of output as firms learn to make products more efficiently, with the decline in cost per doubling of cumulative output known as the learning rate (Arrow, 1962; Wright, 1936).

A vast literature has developed over the decades that applies learning curves to better understand the cost of wind and solar (Junginger and Louwen, 2020; Samadi, 2018; Rubin et al., 2015; Thomassen et al., 2020; Malhotra and Schmidt, 2020). Much of that literature has measured the impacts of a single output factor on prices, using what are known as single-factor models and typically tracing the relationship between cumulative global wind or solar-installed capacity (i.e., learning-by-doing) with the up-front capital cost of wind or solar plants—or, even more narrowly, the cost of wind turbines or solar modules. In practice, however, there are myriad factors that affect costs beyond learning-by-doing; prominent examples include technology R&D, economies of scale, unit size, and exogenous influences such as materials input prices and exchange rate fluctuations. Studies have sometimes used two- or multi-factor learning curves (or other approaches) to account for these additional drivers (Junginger and Louwen, 2020; Elia et al., 2020; Yu et al., 2011; Kavlak et al., 2018; Odam and de Vries, 2020; Elia et al., 2021; Zhou and Gu, 2019; Zheng and Kammen, 2014; Nemet, 2006; Sweerts, Detz, and van der Zwaan, 2020; Lilliestam et al., 2020). As well, there is a recognition that learning rates may not be constant (Van Buskirk et al., 2014; Wei et al., 2017): industries may exhibit temporary periods of accelerated or decelerated learning based on changes in industrial structure, the emergence or relief of resource constraints, and sporadic improvements in specific components of a technology (Junginger and Louwen, 2020; Wei et al., 2017; Ferioli, Schoots, and van der Zwaan, 2009; Yeh and Rubin, 2012).

Notably, most learning curve studies have focused on reductions in the up-front capital costs ($/MW) of plants or subsystems, rather than on reductions in the levelized cost of energy (LCOE, $/MWh)—despite the fact that the wind and solar industries have been more intent on reducing LCOE than on reducing per-unit capital costs. The LCOE represents the levelized cost of all capital and operations and maintenance costs per unit of system output. As per Equation (1), LCOE is a function of not only up-front capital expenditure (CapEx) but also annual operational expenditures (OpEx), plant performance (annual energy production (AEP), inclusive of any performance degradation as plants age), financing costs (in the form of a Capital Recovery Factor that is derived from the real after-tax weighted-average cost of capital (WACC) and plant design life), and income tax rates that feed into the Tax Factor (NREL, 2021; Aldersey-Williams and Rubert, 2019; Short et al., 1995; Rhodes et al., 2017).

(Equation 1) $$\text{LCOE}=\frac{\left(\text{CapEx}\;\text{∗}\;\text{Capital}\;\text{Recovery}\;\text{Factor}\;\text{∗}\;\text{Tax}\;\text{Factor}\right)+\text{OpEx}}{\text{Annual}\;\text{Energy}\;\text{Production }\left(\text{AEP}\right)}$$

Though a lower CapEx often goes hand-in-hand with a lower LCOE, the two do not necessarily always track in the same direction—i.e., higher up-front CapEx can, in some cases, lead to a lower LCOE (e.g., by boosting AEP). Moreover, CapEx is certainly not the only driver of LCOE. Reductions in LCOE can also stem from improvements in plant performance, OpEx, financing costs, and design life (Wiser et al., 2021b; Jones-Albertus et al., 2016; Duffy et al., 2020; Vartiainen et al., 2020), and a small but growing literature has estimated learning rates for (or tracked progress in) some of these other LCOE components, namely OpEx (Steffen et al., 2020; Wiser et al., 2019) and financing costs (Feldman, 2020b; Egli et al., 2018). Some analysts have encouraged the development of (Junginger and Louwen, 2020), and a few studies have even explored (IRENA, 2021; Williams et al., 2017; Wiser et al., 2016), LCOE-based learning rates. Nonetheless, CapEx-based learning remains the norm within the literature, while studies that explore LCOE-based learning are much more scarce—likely because of the difficulty of assembling the requisite data to confidently estimate an historical record of wind and solar LCOE (Santhakumar et al., 2021).

In this study, we apply high-quality granular data to calculate plant-level LCOE for the majority of utility-scale wind and solar PV plants operating in the United States, and then to estimate LCOE-based learning rates across that historical sample. Our contribution to the existing literature is an analysis that brings together a unique combination of five features.

First, we employ plant-level data and recent industry surveys for CapEx, OpEx, capacity factor, WACC, and plant design life to carefully estimate the LCOE for a sizable fraction of all utility-scale wind and solar plants built in the United States—one of the world’s largest renewable energy markets. This bottom-up, plant-level approach provides the granularity needed to control for exogenous influences (as described in the next paragraph), while also enabling us to assess the relative contributions of each of the five key input parameters to historical LCOE reductions over the entire history of both technologies.

Second, we normalize the plant-level LCOE estimates for a variety of exogenous influences unrelated to wind and solar advancements. These normalization factors include regional variation in labor and construction costs, as well as the wind and solar resource, changes in materials prices, the impact of exchange rate movements and import tariffs on imported equipment costs, macroeconomic changes to the WACC, and legislative changes to corporate income tax rates. Carefully normalizing LCOE for these exogenous factors enables us to estimate learning based only on the factors that are actually within the control of the wind and solar industries. Moreover, our approach of normalizing the dependent variable—LCOE—directly avoids some of the statistical issues that can arise when attempting to control for exogenous influences by adding them as independent variables within multi-factor models.

Third, we diverge from the overwhelming majority of the literature by estimating learning rates based on normalized LCOE rather than CapEx. Minimizing LCOE is, and has been, the core design objective in the wind and solar sectors and, as we show, a myopic focus on CapEx learning can understate historical and projected LCOE reductions. Accounting for the many different ways (besides CapEx reduction) in which technology advancement can reduce LCOE adds value not necessarily by reducing the uncertainty surrounding cost projections, but rather by providing greater descriptive value than CapEx-based learning can provide on its own.

Fourth, as a number of previous studies (many not focused on renewable generation) have done (Van Buskirk et al., 2014; Wei et al., 2017), we assess whether historical learning rates have been constant (the typical assumption) or whether they have instead changed over time. As we demonstrate, utility-scale wind and solar LCOE learning accelerated in the final years of our analysis—a finding that shows that learning need not necessarily slow as industries mature.

Finally, we apply this methodology equally to wind and solar in order to comparably assess learning, via a common approach, for two of the central technologies needed for power-sector decarbonization. In contrast, most of the learning curve literature has focused on individual technologies, making cross-technology comparisons difficult.

Although each of these five features of our analysis helps to advance the literature in useful ways, none represents a truly unique contribution on its own, as others have previously incorporated one or more of these elements into their own work. Rather, the value of our work comes from combining these five features within a single analysis. For example, although three somewhat recent studies have similarly focused on LCOE-based learning for wind or solar (as noted above), our work builds on this past research in important ways. Specifically, Williams et al. (2017) also cover the U.S. wind power market, but in contrast to our study, they analyze a shorter historical period, use a different and less-extensive normalization approach, and most importantly do not fully consider advancements in operational costs, project lifetime, and finance—instead choosing to assess learning based primarily on the combined impact of CapEx and performance. Wiser et al. (2016) present four different LCOE-based learning rates for wind energy, including both country-specific and global estimates, but do not independently estimate LCOE—choosing instead to simply derive the learning rates implicit in LCOE trajectories presented in other studies. Moreover, none of those underlying studies sampled by Wiser et al. (2016) normalize for exogenous influences or seek to identify learning change points, and some are quite dated and do not extend to recent years. Finally, IRENA (2021) also estimates LCOE learning across multiple technologies to allow for easier comparison, yet IRENA’s most recent estimates only span the period from 2010 to 2020, and neither assess learning over the full history of each technology nor identify possible change points. Moreover, IRENA (2021) does not normalize for exogenous drivers or comprehensively embed learning across all five factors that influence LCOE. Though each of these three studies that measure LCOE learning provide important contributions to the literature—particularly within the broader context of the myriad other studies that focus more narrowly on CapEx learning—none match the rigor and comprehensiveness of our work.

## Results

### Wind and solar LCOE have declined steeply, but not always smoothly

**Figure 1:**
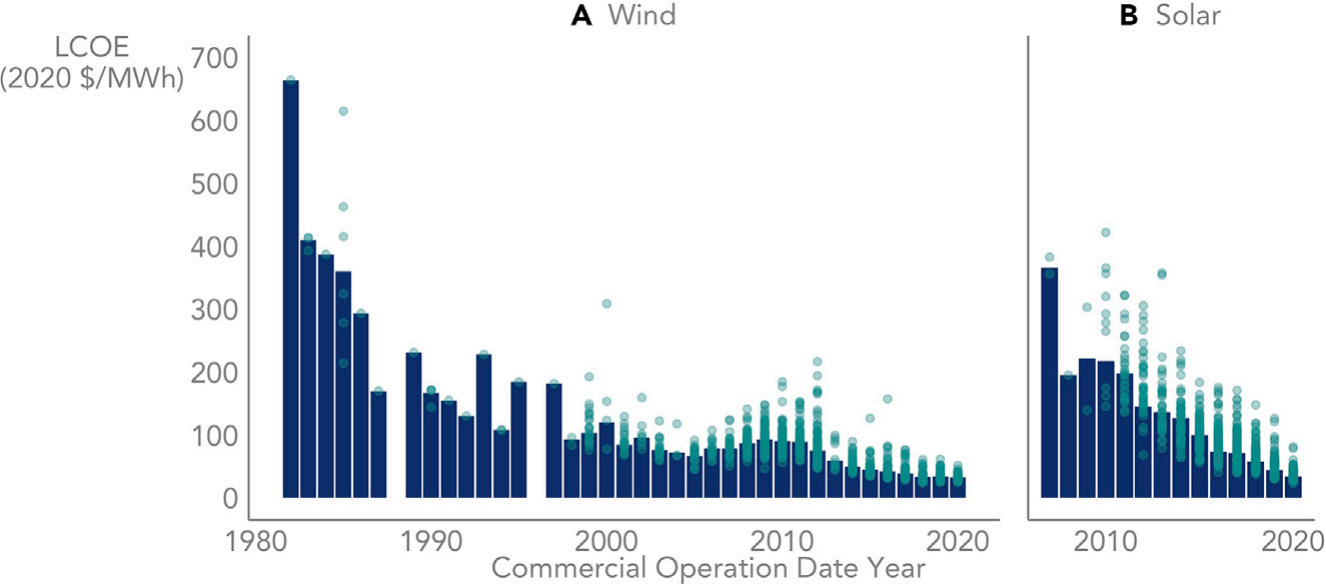
Historical raw (non-normalized) LCOE of utility-scale wind (A) and PV (B) in the United States The circles show individual plant LCOEs based on each plant’s COD year, while the columns show the generation-weighted arithmetic average LCOE, also by COD year. The left figure covers utility-scale wind; the right utility-scale solar. Though the x axes depict time (COD year), within a learning curve framework, it is the increase in cumulative deployment over time, rather than time itself, that drives LCOE lower.

Derived from empirical data described in the STAR Methods, Figure 1 presents the historical LCOE of utility-scale wind and PV plants (defined as onshore wind and ground-mounted PV plants >5 MW) in the United States by the year of each plant’s commercial operation date (COD) and extending back to the inception of each industry in the U.S. There is considerable range in plant-level LCOEs (circles) within any given COD year, but over time (or, more precisely, with greater cumulative deployment), average LCOE (columns) has declined significantly, though not always smoothly. Some of the apparent volatility is due to small sample size in the early years of each market, though exogenous influences unrelated to industry learning also play a role (in the next section, we control for many of these influences).

From an average of $440/MWh over the first few years of the market (1982–1984), wind’s LCOE declined by 93% through 2020 (by which time cumulative global deployment had reached more than 740 GW), to an average of $32/MWh (all costs are expressed in 2020 dollars). Meanwhile, utility-scale PV’s LCOE declined by 85% over a much shorter period—from an average of more than $230/MWh during the first few years of the market (2007–2010) to $34/MWh in 2020 (by which time cumulative global deployment had reached more than 760 GW). Though we portray these LCOE reductions over time, it is worth clarifying that within a learning curve framework, it is cumulative deployment, rather than time itself, that drives the cost reduction. In other words, a decline in LCOE over time is a manifestation, but not a direct measurement, of learning.

Finally, although these LCOEs do not include the benefit of federal tax credits, if we were to factor in these credits, our average LCOE time series for both wind and PV closely track empirical trends in power purchase agreement prices (see Figure S7), bolstering confidence in our underlying plant-level data and LCOE formulation.

### Normalized LCOE controls for exogenous influences to focus attention on industry learning

Before calculating learning rates, we control for a range of exogenous influences on LCOE that do not reflect industry learning; details are found in the STAR Methods, with further sensitivity analyses in Tables S5–S8 and Figure S4. These normalization factors (see Table 1) include regional variation in the quality of wind and solar resources, as well as construction and labor costs; macroeconomic changes to financing costs, exchange rates, and tax rates; changes to materials prices (steel for wind, steel and silicon for PV); and the imposition of tariffs on imported equipment (considered for PV only).

**Table 1:** Normalization factors applied to LCOE components

LCOE Component	Normalization Factor
CapEx	Regional variation in construction and labor costs
	Changes in materials prices (steel for wind and solar, silicon for solar only)
	The impact of exchange rate movements on imported equipment costs
	Import tariffs (solar only)
Capital Recovery Factor	Macroeconomic changes to the WACC
Tax Factor	Legislative changes in federal corporate income tax rates
OpEx	We do not normalize OpEx
AEP	Regional variation in the wind and solar resource

**Figure 2:**
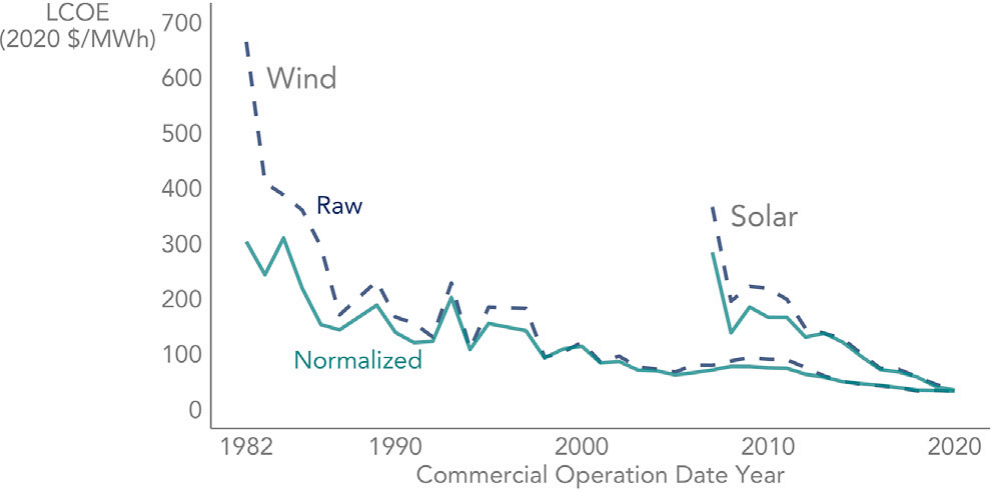
Normalized LCOE controls for exogenous influences unrelated to learning The normalized curves control for changes to regional deployment patterns (via capacity factor, as well as labor and construction costs), exchange rates, finance costs, materials costs (steel for wind, steel and silicon for PV), and income tax rates. The non-normalized (“Raw”) curves are from Figure 1. Both the normalized and non-normalized curves represent generation-weighted arithmetic averages. Though the x axis depicts time (COD year), within a learning curve framework, it is the increase in cumulative deployment over time, rather than time itself, that drives LCOE lower.

Figure 2 compares the raw or non-normalized average LCOE time series from Figure 1 with adjusted versions that normalize the factors listed in the prior paragraph to 2020 levels. To a degree, normalization smooths the time series (more so for wind than solar) and dampens the decline in LCOE over time (i.e., with greater cumulative deployment), as some of the volatility and LCOE reduction in the raw, non-normalized series is attributable to exogenous factors, such as a broader economy-wide drop in interest rates during the 1980s. As a result, the normalized LCOE time series will yield lower learning rates than the original non-normalized series—and should provide a better assessment of industry learning and technology advancement over time (i.e., with greater cumulative deployment).

### Historical LCOE-based learning rates demonstrate distinct epochs

As described in the STAR Methods and further elucidated in the supplemental information (Tables S1 and S2), we use a segmented regression model to discern LCOE-based learning rates for utility-scale wind and PV, and to identify any significant change points in those historical learning rates. We investigated both single-factor (i.e., with cumulative output as the sole regressor) and two-factor (i.e., with both cumulative output and R&D expenditures as regressors) models (Tables S13–S16); the LCOE normalization process described earlier largely obviated the need to explore multi-factor models with more than these two regressors. We also explored several different interpretations of cumulative output, including various permutations of U.S. versus global, utility-scale versus total, and energy versus capacity (see Tables S9–S12). In general, the single-factor models outperformed the two-factor models in terms of explanatory power. Similar to other cases of two-factor models (Yeh and Rubin, 2012), cumulative wind and solar output are highly collinear with R&D expenditures, thus undermining the application of a two-factor approach in our study. Further, global total capacity generally outperformed (or at least did not significantly underperform) other interpretations of cumulative output (though in many cases, differences are subtle, signifying a relatively robust and stable model—see Tables S9–S12). Henceforward, all results are based on our preferred specification using normalized LCOEs in a single-factor model using global total capacity.

**Figure 3:**
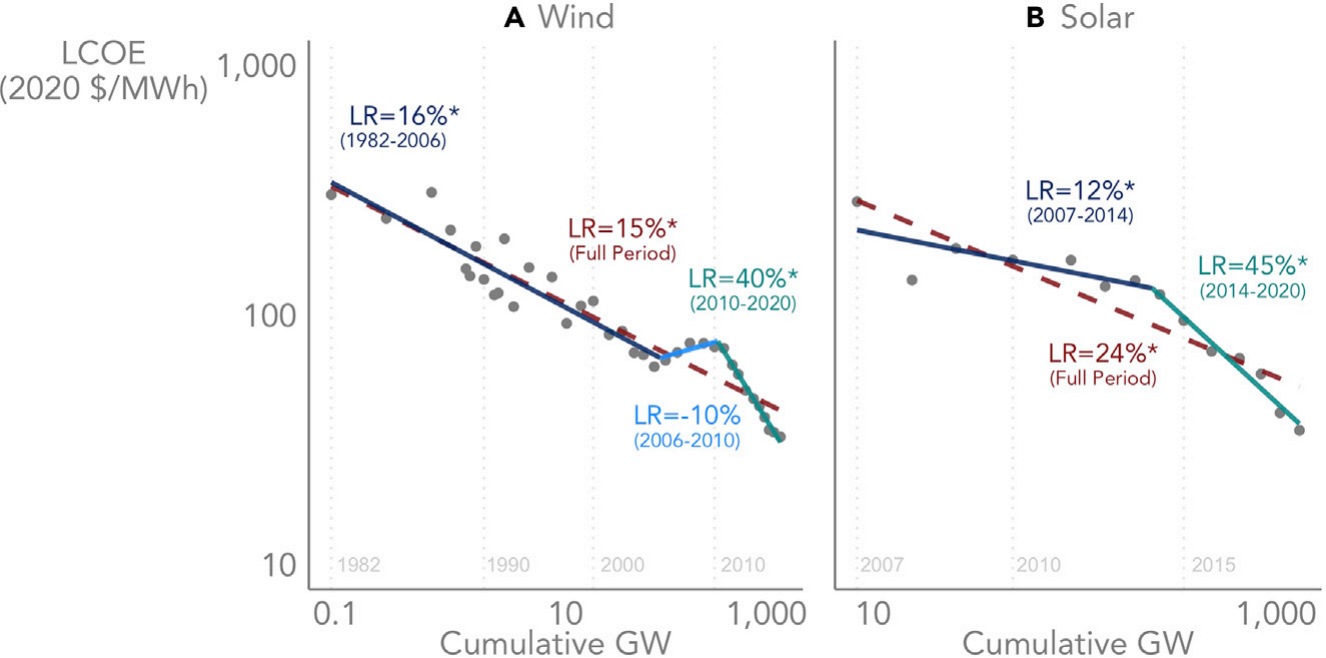
LCOE-based learning curves for utility-scale wind (A) and PV (B) exhibit distinct epochs The learning curves are based on the generation-weighted arithmetic average normalized LCOE from Figure 2 (dots), and the axes are logarithmic. An asterisk indicates statistical significance (p < 0.05).

The segmented regression identified two change points occurring around 2006 (66.8 GW) and 2010 (210.6 GW) in the case of wind, and a single change point occurring around 2014 (162.8 GW) in the case of solar (Figure 3). Wind’s epoch of negative learning from roughly 2006 to 2010 reflects an inflationary period in which the U.S. dollar weakened (increasing the cost of imported wind turbine components) while commodities and fuel prices, as well as wind turbine manufacturers’ warranty and labor costs, all rose. Moreover, higher natural gas prices and other factors may have provided cover for wind turbine manufacturers to raise prices even more, in an effort to rebuild their flagging profit margins (Das et al., 2020). From an LCOE perspective, this period of negative learning could also partly reflect a change in federal policy from 2009 to 2012, when—due to a shortage of tax equity following the financial meltdown of 2008—wind projects were able to elect an investment-based cash grant (equal to ∼30% of plant costs) in lieu of the production-based production tax credit (again, we do not include tax credits in our LCOE calculations). Many wind developers took advantage of the cash grant to complete the more costly and/or less energetic projects in their development pipelines, thereby contributing to a higher average LCOE over this period. Our LCOE normalization method controls for some, but not all, of these potential drivers of negative learning—for example, we were not able to account for changes in manufacturer or owner profitability, which can result in varying cost-price markups over time.

For both technologies, the most recent learning epoch (i.e., ∼2010–2020 for wind and ∼2014–2020 for PV) features the highest learning rates of 40% for wind and 45% for solar, implying a 40% and 45% reduction in LCOE for each cumulative doubling of global total wind and solar capacity, respectively. Of course, wind’s recent accelerated learning rate of 40% might potentially be viewed as merely compensating for the negative learning (−10%) exhibited from roughly 2006–2010, with the two periods essentially canceling one another. Moreover, it is difficult to predict whether, or for how long, the recent accelerated learning rates will persist into the future (Figure S3 of the supplemental information explores the effect of different assumptions about the persistence of accelerated learning on LCOE projections). Nevertheless, the fact that both wind and PV exhibit accelerated learning more recently is notable and concurs with previous research showing that learning need not necessarily moderate as technologies and industries mature. Temporary periods of accelerated learning could reflect adjustments in industrial structure as a technology matures, such as periods of accelerated market entry and competition (Wei et al., 2017). Over the full histories of each technology, the estimated learning rates are 15% for wind (18% if based entirely on raw, non-normalized data) and 24% for utility-scale PV (27% if based on raw, non-normalized data).

The LCOE-based learning rates estimated here for wind are broadly comparable to three other recent studies that assess LCOE learning. The LCOE-based learning rate from Williams et al. (2017), at 10%, is somewhat lower than our estimate of 15%, but Williams et al. (2017) analyze a shorter historical period, use a different normalization approach, and do not fully consider advancements in operational costs, project lifetime, and finance. Wiser et al. (2016) present four historical LCOE-based learning rates for wind, ranging from 10.5% to 18.6%. Analyzing a more recent period of 2010–2020, IRENA (2021) estimates a learning rate for land-based wind of 32%, roughly consistent with our finding of recent accelerated learning. Both Williams et al. (2017) and IRENA (2021) also emphasize the importance of LCOE-based learning rates for wind, finding much lower rates of learning if solely evaluating CapEx improvements. Finally, though comparable literature on LCOE learning for utility-scale solar is scarce, IRENA (2021) estimates a 39% rate over the 2010–2020 period, comparable to our 45% estimate in the most recent epoch.

### Learning rates associated with individual LCOE components shed light on LCOE drivers, but misrepresent overall LCOE advancement

**Figure 4:**
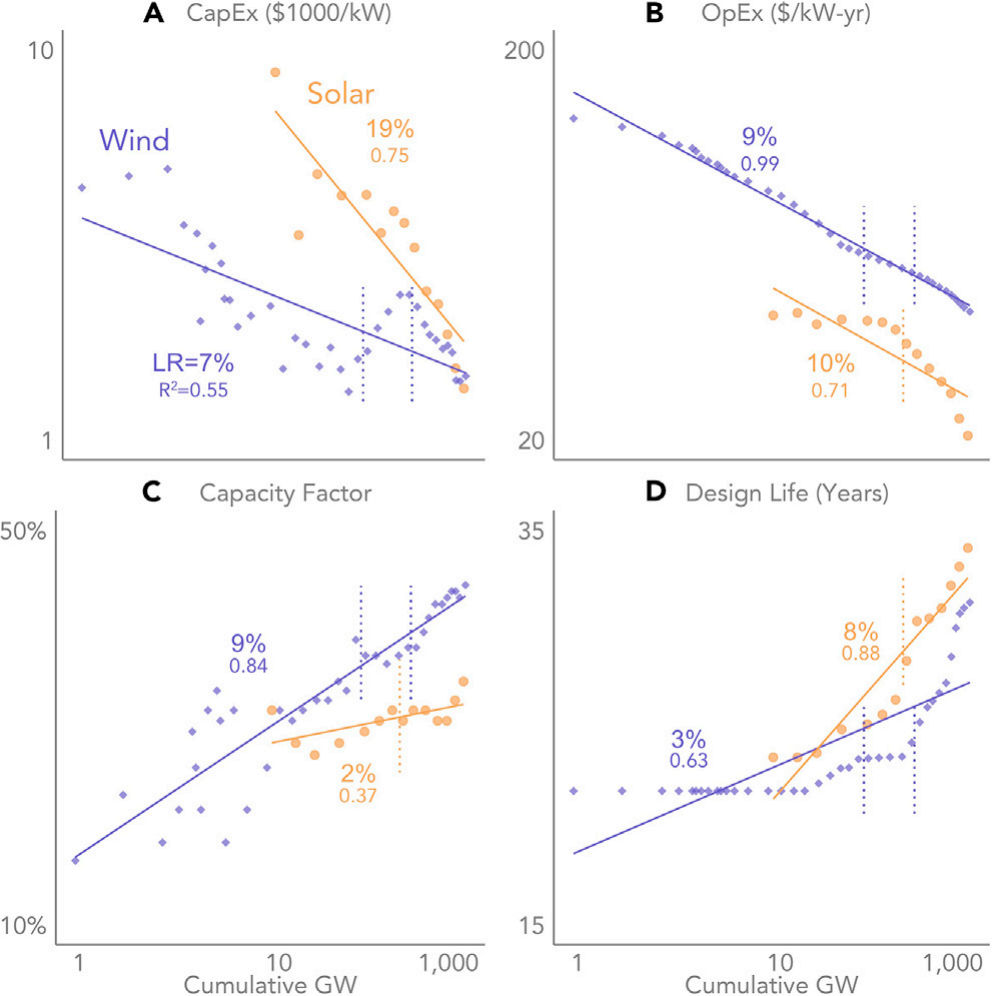
Learning curves for individual components of LCOE shed light on LCOE drivers Here, we present only full-period learning rates; the dashed vertical lines correspond to the LCOE change points identified in Figure 3 (see Tables S1–S4 and Figures S1 and S2 of the supplemental information for an analysis of change points). All learning rates are expressed with positive signs, even though CapEx (A) and OpEx (B) decline while Capacity Factor (C) and Design Life (D) increase (all four trends contribute to lower LCOE). The CapEx and Capacity Factor data are normalized. The axes are logarithmic. All four LCOE components exhibit correlations with cumulative output that are consistent with learning, as indicated by the strong R^2^ values depicted in the figure.

Figure 4 presents learning rates for individual components of LCOE: CapEx, OpEx, capacity factor, and design life. Here, we focus on full-period learning rates, without looking for change points (though the dashed vertical lines in Figure 4 do show the overall change points identified in Figure 3), both in the name of expediency and so that we can more easily compare our component learning rates (e.g., for CapEx) to past estimates from the literature. We exclude finance costs in this assessment as our approach did not yield evidence of wind- or solar-specific learning in the cost of finance, at least when normalized for macroeconomic influences.

When focusing solely on CapEx (as has most of the literature), our learning rates of 7% for wind and 19% for PV (both based on normalized CapEx data) are reasonably consistent with past literature (Rubin et al., 2015). Yet CapEx tells only part of the story. For wind, CapEx has a full-period learning rate (7%) that is only half as large as our full-period normalized LCOE learning rate of 15%. For utility-scale PV, the difference is not nearly as stark—19% for normalized CapEx versus 24% for normalized LCOE—yet is nevertheless directionally consistent with wind and tells much the same story: that LCOE learning reflects and benefits from learning in each individual LCOE component, and not just CapEx. Moreover, precisely because LCOE depends on more than just CapEx as an input, a 7% CapEx learning rate does not have the same effect on LCOE as does a 7% LCOE learning rate—i.e., a 7% decrease in CapEx results in a lesser-magnitude decrease in LCOE. As such, the singular focus on CapEx that pervades much of the learning curve literature is misleading, and results in an incomplete understanding of technology advancement, perhaps leading to underestimation of future wind and solar LCOE reductions (Figure S6 explores this idea further).

**Figure 5:**
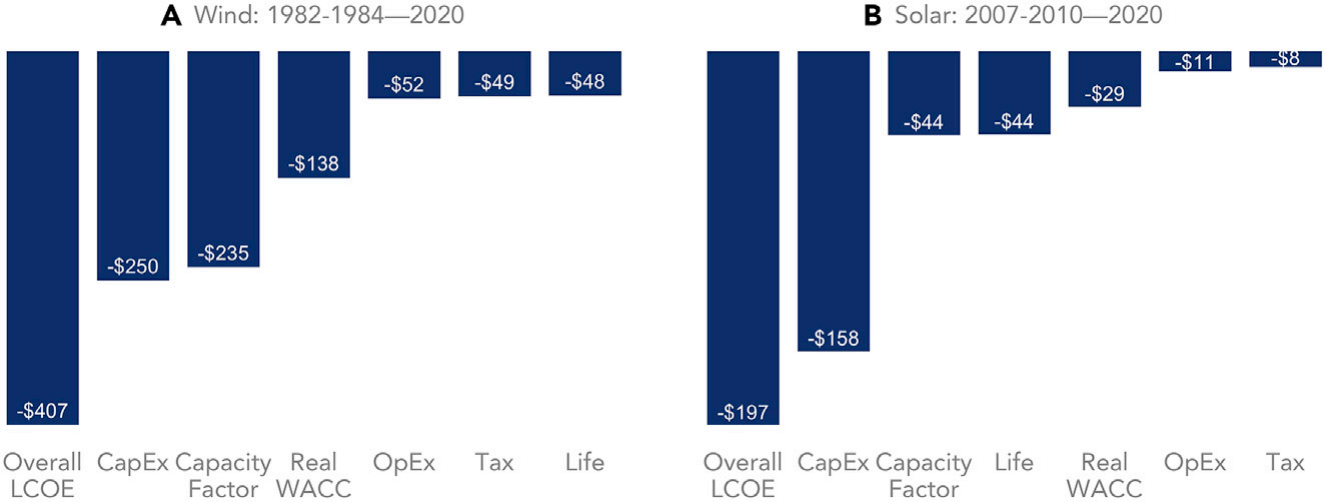
CapEx has been the largest, but not the only, driver of LCOE reduction The graphs show the decline in LCOE through 2020 from a starting point of 1982–1984 (average) for wind [A] and 2007–2010 (average) for utility-scale PV [B] (these multi-year generation-weighted arithmetic average starting points compensate for small sample size in the early years of each market). Both graphs present non-normalized data. Interactions between the components prevent the numbers from being additive; the purpose is to show the relative contribution of individual LCOE components.

Figure 4 also helps shed light on the learning rate change points identified in Figure 3, and why LCOE learning has accelerated through 2020. For wind, expectations for design life have increased sharply in recent years, while capital costs have dropped significantly (though again, one might argue that the sharp decline in capital costs may simply be in response to the increase from 2006 to 2010). More subtly, and as discussed further below Figure 5, the recent (i.e., following the latest change point) acceleration in capacity factor gains and OpEx declines likely reflects wind turbine scaling (i.e., greater capacity, larger rotors, and taller towers) over this period. For PV, recent capital and operating cost declines have accelerated with rapid deployment through 2020, as has the increase in design life. See the supplemental information (Figures S1 and S2; Tables S3 and S4) for further exploration into the drivers of learning change points.

Finally, Figure 5 reinforces the message that LCOE learning is the result of advancements in multiple components by graphing the relative contribution of individual LCOE components to overall LCOE decline since the start of each market. Given small sample size in the early years of each market, we use the multi-year generation-weighted arithmetic average LCOE from 1982 to 1984 for wind and from 2007 to 2010 for PV as our starting points. For each individual LCOE component (e.g., CapEx, capacity factor, etc.), we then change the starting point value for that component to its generation-weighted arithmetic average 2020 (i.e., end point) value, and use the LCOE equation to calculate the resulting reduction in LCOE, which we attribute to that individual component.

For wind, declining CapEx has been the largest driver, followed by increasing capacity factor and a decline in financing costs (note that Figure 5 presents only non-normalized data; normalized financing costs are not as much of a contributor, as much of the decline in WACC over this period—and particularly during the 1980s—has been economy-wide and not specific to wind or solar). The fact that wind’s capacity factor is a slightly smaller contributor than its CapEx in Figure 5 despite having a slightly higher learning rate than CapEx in Figure 4 is not contradictory, but rather highlights CapEx’s greater influence on wind’s LCOE in general. For utility-scale PV, declining CapEx has dominated.

Because of interactions among the components shown in Figure 5, one cannot simply sum the individual component LCOE reductions to arrive at the overall LCOE reduction. For example, higher CapEx devoted to wind turbine scaling can reduce LCOE from multiple angles: by reducing balance-of-plant CapEx (e.g., fewer foundations and access roads per MW), by reducing OpEx (e.g., fewer tower climbs per MW), and by boosting capacity factor (through taller towers accessing better winds, and via larger rotors relative to turbine capacity). Similarly for solar, higher CapEx spent on single-axis tracking (rather than fixed-tilt racking) or more-reliable modules and inverters might again lead to a lower LCOE via a higher capacity factor, lower OpEx, and a longer useful life. This interactivity between CapEx, capacity factor, OpEx, and design life highlights complications that can arise from relying solely on component-based (rather than LCOE-based) learning, and especially calls into question the past literature’s affinity for focusing exclusively on CapEx-based learning.

### Relative uncertainties suggest use of full-period learning rates for LCOE projections

**Figure 6:**
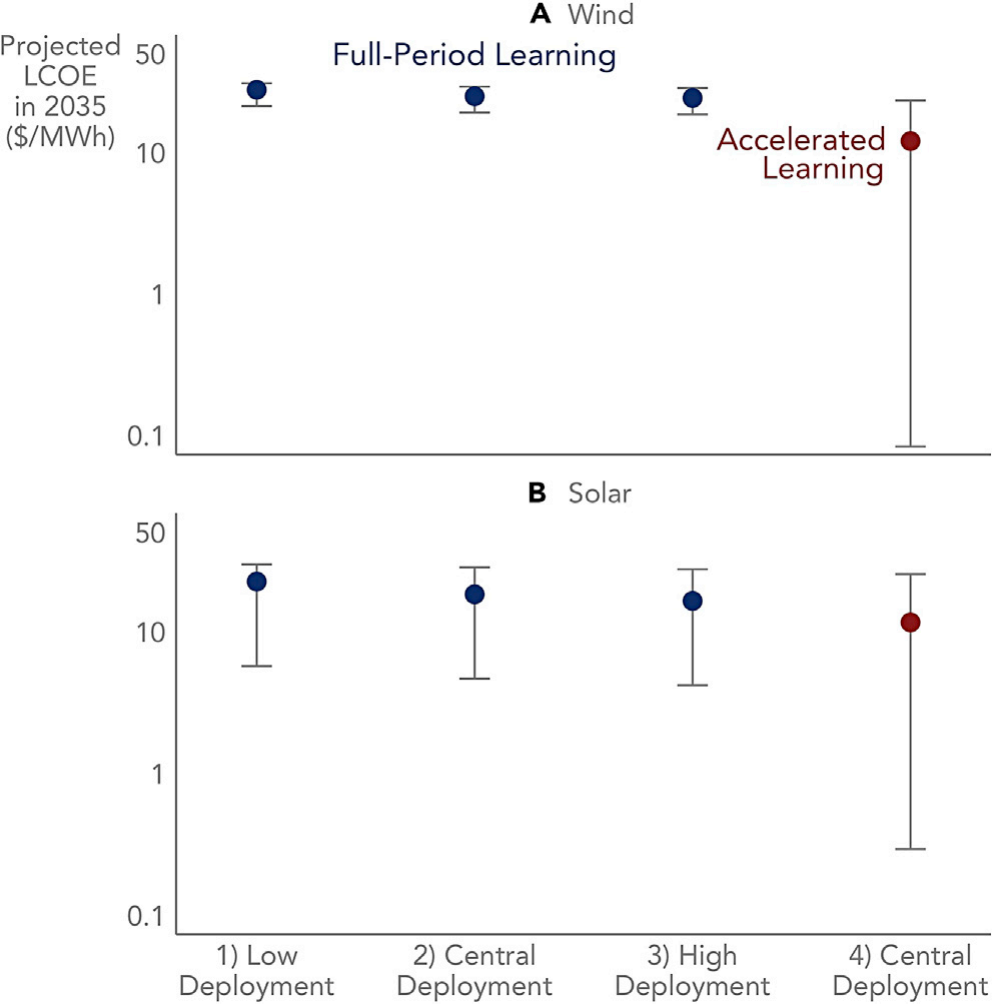
Sources of uncertainty in LCOE projections (A and B) For both wind (A) and solar (B), the plot shows four different projected LCOE point estimates and 95% confidence intervals for the year 2035 based on full-period (constant) learning and low, central, and high estimates of future deployment (numbers 1–3) and accelerated learning (based on the most recent epoch) using central estimates of future deployment (number 4).

There are at least three sources of uncertainty when using learning rates to project future cost trajectories: uncertainty over which learning rate to choose when learning is non-constant, uncertainty surrounding the chosen learning rate itself, and uncertainty over to which future deployment projections to apply the chosen learning rate. [An additional overarching uncertainty is whether to project LCOE based on historical LCOE-based learning rates—as we do in this paper—or to instead project the individual components of LCOE based on their respective historical learning rates and then calculate LCOE from those projected components; Figures S5 and S6 of the supplemental information explore this choice.] Figure 6 depicts these three sources of uncertainty by projecting LCOE point estimates for the year 2035, along with their associated forecast errors using the method described in STAR Methods. The projections are based on low, central, and high estimates of future deployment (see Figure S8), using both full-period (constant) learning rates and the accelerated learning rates from the most recent epochs.

In the case of wind, projections based on the full-period learning rate yield relatively tight and consistent LCOE estimates (due primarily to wind’s longer historical time series of 39 years), and there is far greater uncertainty tied to the choice of learning rate itself—i.e., the lower full-period learning rate yields a significantly different LCOE point estimate than the higher more recent learning rate. In the case of solar, the point estimates based on full-period learning are inherently more uncertain due to the shorter time series (14 years), though the choice of learning rate is also a key factor in future cost projections. For both technologies, the projections based on accelerated learning in the most recent epochs are highly uncertain given the short timespans of each epoch: 11 years for wind and 7 years for solar. Furthermore, in both cases, the likely persistence of recent accelerated learning into the future is unclear (e.g., supply chain disruptions related to the Covid-19 pandemic have already pressured LCOE higher in 2021 and early 2022). Hence, while the segmented, non-constant learning curves from Figure 3 provide useful insights into historical cost reductions, for the purposes of projecting future LCOE with more certainty, we focus solely on the full-period learning rates.

**Figure 7:**
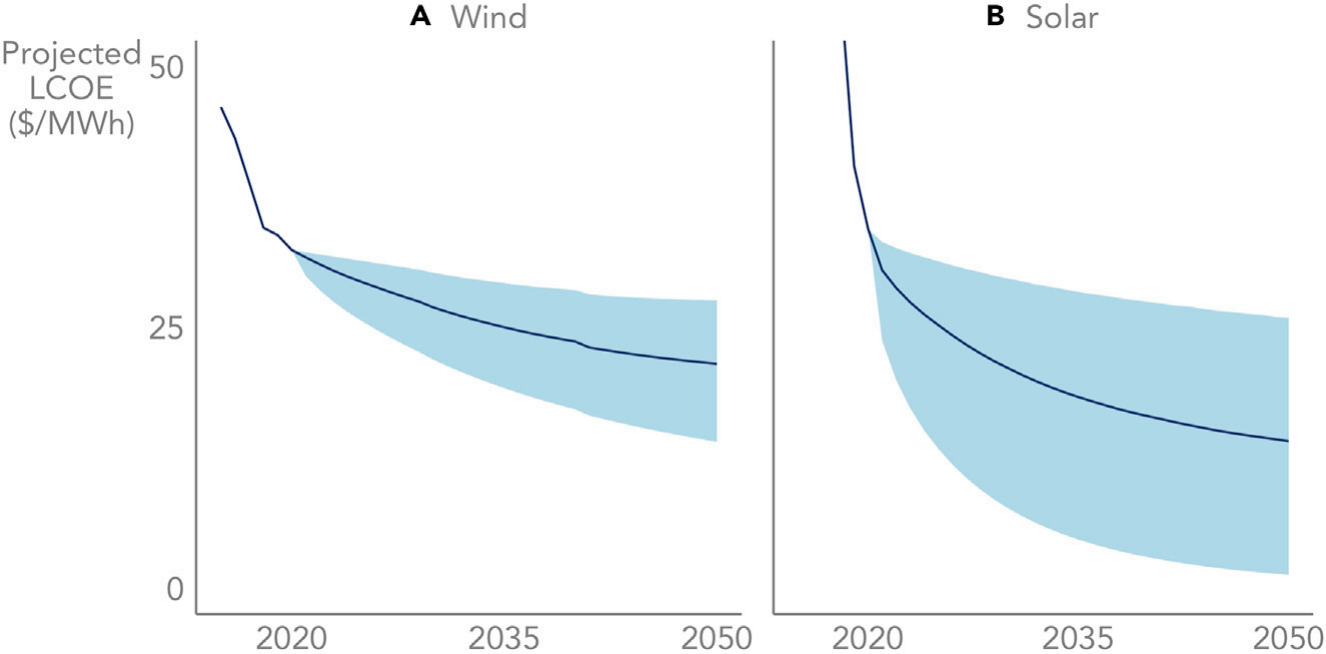
Projected LCOE based on full-period learning rates For both wind (A) and solar (B), LCOE projections are based on the full-period learning rates and central estimates of future deployment (IEA, 2020; EIA, 2019; DNV, 2020; BloombergNEF, 2020; IRENA, 2020; Wood, 2020). The lines represent point estimates and the bands represent 95% confidence intervals based on forecasting methods described in the STAR Methods.

Figure 7 depicts the LCOE projections based on full-period learning rates and central estimates of future deployment (IEA, 2020; EIA, 2019; DNV, 2020; BloombergNEF, 2020; IRENA, 2020; Wood, 2020). We assume that forecast uncertainty increases over time, such that long-term projections are inherently less certain than near-term projections (see the STAR Methods). Long-term forecasts are particularly uncertain in the case of solar, largely due to the shorter duration of that historical time series. Nonetheless, both projections accord with broader forecasts for ongoing costs reductions in wind and solar. In the case of wind, the 15% full-period learning rate suggests that LCOE will decline by around 23% by 2035, or as much as 41% based on the lower bound of the 95% confidence interval. In the case of solar, the 24% full-period learning rate suggests a 47% LCOE reduction by 2035, or as much as 86% based on the lower bound of the 95% confidence interval.

As a point of comparison, we benchmarked the LCOE projections shown in Figure 7 against NREL’s 2021 Annual Technology Baseline (ATB), which provides a bottom-up, engineering-based projection of the LCOE of various technologies through 2050 (NREL, 2021). The 2021 ATB finds that the LCOE of both utility-scale PV and wind could fall to ∼$10/MWh by 2050 at the best sites. These ATB projections are well below Figure 7’s 2050 point estimates of $14/MWh for utility-scale PV and $21/MWh for wind (based on their respective full-period learning rates). That said, the lower bound of solar’s 95% confidence interval in Figure 7 does dip below $10/MWh—to just $1.3/MWh—by 2050, which is more of a reflection of the high degree of uncertainty that far out in the future than it is of a likely outcome.

Given the wide confidence intervals—particularly for solar—shown in Figure 7, it is worth reiterating that the value of our analysis is not in reducing the uncertainty surrounding LCOE projections. Rather, the value of our analysis is in improving the ability of learning models to describe technology advancement as it is perceived and measured within the wind and solar industries—i.e., in terms of LCOE as a whole, rather than in terms of individual LCOE components, such as CapEx. While component-level learning is, of course, part of LCOE-based learning, it is not sufficiently descriptive on its own. For example, if we apply the 7% wind CapEx learning rate from Figure 4 to the historical 1982 wind LCOE, we arrive at a projected 2020 LCOE that is almost four times higher than the actual historical 2020 wind LCOE. In contrast, applying the 15% LCOE-based learning rate from Figure 3 to the historical 1982 wind LCOE yields the actual historical 2020 wind LCOE, by definition. Similarly, Figure S6 in the supplemental information shows that CapEx-based and LCOE-based learning rates yield vastly different LCOE projections through 2050. Historically, LCOE—not CapEx or anything else—has been the core optimization variable in the wind and solar sectors, so it stands to reason that we should measure technological progress in terms of LCOE-based learning. Doing so enables us to account for the many different ways in which technology advancement can improve LCOE.

## Discussion

Lower costs for wind and solar can facilitate power-sector decarbonization. Maintaining strong pressure for cost reduction may be especially important given the many institutional challenges that can slow deployment (Cherp et al., 2021) and the increasing recognition that the grid-system value of wind and solar tends to decline as penetrations increase (Das et al., 2020; Sivaram and Kann, 2016). So far, wind and solar cost reductions have kept pace with value declines, but future technology advancement will dictate whether these trends continue (Millstein et al., 2021).

Though learning curves are not the only tool for predicting future cost reductions, the concept of endogenous learning is well established, and learning curves offer a practical and often-used means of projecting future outcomes. Moreover, other cost-projection methods have not proven demonstrably superior. This paper develops well-grounded learning estimates for utility-scale wind and solar that advance the existing literature. What do we conclude based on our assessment?

We conclude that the details surrounding learning curve specification matter. The wind and solar industries have rightly focused attention on minimizing LCOE; CapEx is a crucial input into LCOE, but not the only one. As such, a myopic focus solely on CapEx learning ignores other opportunities for LCOE reduction, thereby leading to an incomplete understanding of technology advancement, which in turn can dampen future cost reduction and therefore deployment expectations. In contrast, LCOE-based learning provides a more holistic and nuanced assessment, by considering the full range of technology advancement and cost-reduction levers. Moreover, normalizing LCOE components to remove exogenous influences that have no bearing on learning can lead to a more accurate assessment of industry advancement—as well as meaningfully different learning estimates. At a minimum, our work suggests that the literature could profit from greater attention to LCOE-based learning, and to careful learning curve specifications that control for exogenous cost influences.

Consistent with previous studies, we also find that learning—and particularly LCOE learning, which benefits synergistically from learning among its individual components—need not be constant and also need not always moderate as technologies mature. Our work demonstrates not only different epochs during which costs have progressed on distinct paths, but also a recent period through 2020 of accelerated cost reduction for both wind and solar. We qualitatively discussed some of the possible drivers for this accelerated learning, but understanding the underlying technology, industry, and policy motivators for these developments remains an area of future research. Overall, our work confirms that learning is not a fixed, static process, but is instead one that can lead to periods of accelerated or decelerated change. The use of segmented learning curves is one means of illustrating these shifts.

These findings have implications for the energy and climate policy, planning, and modeling communities. First, it is important that these communities account for non-constant learning. There is evidence that common energy-sector planning and integrated assessment models have not kept pace with the recent declines in wind and solar costs, and so may be understating the deployment potential for these technologies (Xiao et al., 2021; Grubb et al., 2021b), though recent research on historical growth rates suggests that other model features may result in overstated potential (Cherp et al., 2021).

Second, these same models do not always account for endogenous learning—sometimes preferring to input exogenous cost trajectories (Krey et al., 2019; Grubb et al., 2021a; Cole et al., 2020). In other cases, planners and modelers assume that learning rates decline as cumulative deployment increases (EIA, 2021). In contrast, our results add to the body of literature that suggests that endogenous learning is a real phenomenon, and show that learning need not moderate with industry maturity. While the proper treatment of endogenous learning in energy and climate models is a complicated matter, at a minimum, our results suggest further effort in this direction is warranted—adding to other areas of possible model improvement (Grubb et al., 2021a, 2021b).

Finally, we show that an exclusive focus on CapEx learning can understate the LCOE reduction potential for utility-scale wind and solar energy. To the extent that energy and climate planning models employ endogenous learning, we encourage a move away from the historical focus on CapEx learning (Krey et al., 2019; EIA, 2021) and toward a more holistic understanding of the multiple levers for LCOE-based learning. Doing so is likely to yield lower future cost estimates than currently assumed in many models.

## Limitations of the study

Learning curves have been criticized for a number of reasons. The most common criticism—with which we very much agree—is that learning curves often focus on capital costs and ignore other means of reducing generation costs, while simplifying the many drivers of cost reduction (Junginger and Louwen, 2020; Ferioli, Schoots, and van der Zwaan, 2009). Our analysis sidesteps this criticism by focusing on LCOE rather than capital costs, and by controlling for exogenous influences that are unrelated to learning. Nevertheless, our study is not immune to some of the other criticisms commonly levied at learning curves. For example, using historical data to project future outcomes assume that future trends will replicate past ones—an assumption that may or may not hold (Nordhaus, 2014). And the direction of causality is not clear: deployment can reduce costs, but so too can reduced costs induce deployment (Grubb et al., 2021a). Finally, the diversity of specifications and underlying data has resulted in a wide range of learning rates in the literature, creating challenges for practical application (Junginger and Louwen, 2020).

Even with well-specified learning models, it is important to acknowledge the deep uncertainties in future cost projections. Though we believe the normalized LCOE-based learning specifications presented in this paper represent an improvement over much of the existing literature, the resulting learning rates (and confidence intervals around them) should be viewed with a critical eye. Specifically, our analysis identified one or more change points in historical learning for both wind and solar, suggesting that additional shifts are possible, and perhaps even likely, in the future. Moreover, our analysis also suggests that non-constant learning increases forecast uncertainty, as the existence of one or more learning change points reduces the length of time over which each learning rate is measured, and the likely persistence of the most-recent learning rate into the future is uncertain. For these reasons, we use only full-period learning rates for the purposes of projecting future costs, though even projections based on full-period learning will yield wide confidence intervals in the long term when allowing forecast uncertainty to expand with time. Finally, although we normalized historical LCOE before calculating learning rates, the exogenous influences that we excised from the historical data will no doubt continue to divert LCOE from a purely learning-related path going forward (e.g., witness the current supply chain disruptions caused by the Covid-19 pandemic, which have pressured both wind and solar LCOE higher in 2021 and early 2022). For these and other reasons, an appreciation for the uncertainties in future costs is appropriate, regardless of the underlying modeling or planning approach.

The geographic focus of our analysis is limited to the United States. That said, our findings (with associated uncertainties in hand) are potentially more broadly applicable to other markets, for a few reasons. The historical period of study—since 1982 for wind and since 2007 for solar—roughly spans the full modern-day history of these two technologies in utility-scale applications around the world. Both technologies rely heavily on global, rather than domestic, supply chains. Although U.S. markets have contributed a significant fraction of worldwide deployment of both technologies, it is global, rather than domestic, deployment that drives learning in our model. Finally, and most importantly, our learning rates are based on normalized LCOEs that do not include the effect of tax credits or other U.S.-specific incentives, and that control for exogenous influences that are unrelated to learning. These normalization factors include a number of U.S.-specific variables—like exchange rate impacts, import tariffs, construction and labor costs, tax rates, and resource strength—that, once controlled for, make our normalized LCOE time series less country-specific and more global in nature. Even still, applying our methodology to similar data from other countries would nevertheless be valuable.

Of course, accurately projecting LCOE at the busbar—whether via learning rates or some other means—is only one of the many challenges with modeling the deployment potential of new technologies. Equally important are various institutional and social deployment barriers, as well as the grid value of generation resources. Moreover, the future potential of variable renewable resources like wind and solar will likely hinge, to a large extent, on the cost and value of other enabling technologies such as energy storage and new transmission. Even as the LCOE of wind and solar continues to decline, system-wide cost reductions will nevertheless be tempered by a growing need for new storage and transmission in order to integrate higher penetrations of wind and solar. As such, it is important to keep in mind that our analysis narrowly focuses on just one element—projected LCOE—of the full suite of economic and institutional considerations that will ultimately determine the deployment of utility-scale wind and solar power in the future.

## STAR★Methods

### Key resources table

**Table:** 

REAGENT or RESOURCE	SOURCE	IDENTIFIER
**Deposited data**		

Raw and analyzed data	This paper	N/A

### Resource availability

#### Lead contact

Further information and requests for resources should be directed to and will be fulfilled by Mark Bolinger (mabolinger@lbl.gov).

#### Materials availability

This study did not generate new materials.

### Method details

#### Estimating actual LCOE

We adopt the LCOE formula (Equation 1) used in NREL’s *Annual Technology Baseline* (NREL, 2021), and apply it to a large sample of individual utility-scale wind and solar plants. Equation (1) requires a number of inputs, including capital and operating costs (CapEx and OpEx), financing costs (in the form of a Capital Recovery Factor that is derived from the real after-tax WACC and plant design life), income tax rates (that feed into the Tax Factor), and annual energy production (AEP, derived from levelized capacity factor data, as described below). For the two most important of these inputs—CapEx and AEP—we rely on empirical plant-level data from a sample of 908 wind plants totaling 106.5 GW_AC_ and with COD years ranging from 1982 to 2020, as well as 822 utility-scale PV plants totaling 33.7 GW_AC_ and with COD years ranging from 2007 to 2020. Only wind and solar plants with capacities >5 MW_AC_ are included in our sample. All other inputs vary by calendar year (rather than by individual plant) and we apply them uniformly by COD year to all plants in our sample.

Empirical plant-level CapEx and capacity factor data through 2020 (as well as data on various plant characteristics, such as installed capacity and COD) are from Wiser et al. (2021a) for wind and Bolinger et al. (2021) for solar. As necessary, we extend capacity factors beyond calendar year 2020 for those plants that had not yet reached the end of their design life by that year. In such cases, the extension beyond 2020 to the full term of the design life applies the appropriate representative performance degradation rates for new and old plants from Hamilton et al. (2020) for wind and Bolinger et al. (2020a, 2020b) for solar. We then levelize the resulting capacity factor time series over each plant’s design life (which varies by COD year), using the appropriate real after-tax WACC (described below) as the discount rate.

The remaining LCOE inputs—i.e., OpEx, financing costs (including WACC, inflation rates, and plant design life), and income tax rates—are not plant-specific, but rather vary only by calendar year, and are applied uniformly by COD year to all plants in our sample. Operating costs are from Wiser et al. (2019) for wind and Wiser et al. (2020) for solar. The nominal after-tax WACC draws upon representative debt interest rates and expected equity returns (both expressed on an after-tax basis) that vary yearly (Wiser et al., 2021a; Intercontinental Exchange, 2021; Kahn, 1991, 1995; Wiser and Kahn, 1996; Harper et al., 2007; Karas, 1994), as well as a constant debt/equity ratio of 70%/30% over time for both wind and solar (Feldman, 2020a; NREL, 2021). We use estimated inflation expectations going back in time from the Federal Reserve Bank of Cleveland (2021) to convert the nominal after-tax WACC to real dollar terms. Finally, we apply design lives from Wiser and Bolinger (2019) for wind and Wiser et al. (2020) for solar to convert the real after-tax WACC to the capital recovery factor required for the LCOE formula. Combined federal and state income tax rates for each COD year are based on the maximum federal corporate income tax rate at the time and an assumed constant 5% state income tax rate, with state taxes assumed to be deductible from federal.

Though our LCOE calculations do not include the benefit of federal or state tax credits (though Figure S7 of the supplemental information presents an LCOE time series that does include federal tax credits), they do include the benefit of accelerated depreciation for federal tax purposes, which is permanently available to wind and solar plants within the U.S. tax code. Specifically, we apply the mid-year convention of the 5-year Modified Accelerated Cost Recovery Schedule to our plant-level capital cost estimates, and then discount this 6-year stream using the nominal after-tax WACC, per Short et al. (1995). This, in turn, feeds into the Tax Factor input to the LCOE equation.

The process described above yields LCOE estimates for each plant in our wind and solar samples. We derive LCOE averages for each calendar year by calculating the generation-weighted arithmetic average LCOE among all plants whose COD year equals the calendar year.

#### Normalization of LCOE for exogenous influences

The process described above results in the estimation of an LCOE time series that is largely empirical and therefore reflects the influence of both learning and non-learning factors. Rather than use this time series to estimate learning rates, we instead control for a number of exogenous, non-learning factors that can influence capital costs, capacity factor, and finance costs (we also normalize income tax rates) in order to sharpen the focus on learning. Table 1 lists the normalization factors that we apply to four of the five LCOE components from Equation (1) (we do not normalize OpEx), while the text that follows provides detail on the implementation of each normalization factor. Tables S5–S8 and Figure S4 show the impact of these normalization factors on the full-period learning rates of wind and solar.

We normalize the capital costs of wind and solar plants to COD year 2020 levels by controlling for (1) regional variations in construction and labor costs, given shifting deployment among regions over time; (2) raw materials prices (steel for wind, steel and silicon for PV); (3) exchange rate movements, given that some equipment is imported into the United States; and (4) import tariffs (solar only).

We control for regional variations in construction and labor costs by assigning each wind and solar plant a regional cost multiplier based on its location (from https://atb.nrel.gov/electricity/2019/regional-capex.html). We then multiply each plant’s $/W capital cost by the ratio of the generation-weighted arithmetic average regional multiplier across all plants with a 2020 COD year (the numerator) to the plant’s own assigned regional multiplier (the denominator). This normalizes each plant’s capital costs to the 2020 average mix of plant locations. This approach implicitly assumes that these regional multipliers have not changed over time—likely an imperfect assumption.

We control for changes in steel (for both wind and solar) and silicon (solar only) prices over time using a representative kg/kW mass for each material/technology pairing—140 kg of steel per kW wind (Vestas, 2006; Garrett and Ronde, 2011a, 2011b; Razdan and Garrett, 2015a, 2015b, 2018a, 2018b), 95.9 kg of steel per kW solar (Smith and Margolis, 2019), and 2.7 kg of silicon per kW of solar (Smith and Margolis, 2019)—and an annual $/kg price time series for each material (Bernreuter Research, 2021; U.S. Bureau of Labor Statistics, 2021). For each plant and material pairing, we multiply the representative mass times the price in the COD year to arrive at a $/kW cost. We subtract from that cost the generation-weighted average material cost across all plants with a 2020 COD. Finally, we subtract the resulting $/kW delta from each plant’s capital cost, in essence normalizing all capital costs to 2020 steel and silicon prices.

Although steel and silicon are only two of many different materials used to build wind and solar plants, research suggests that these two account for the majority of overall materials costs (Bolinger and Wiser, 2012; Smith and Margolis, 2019). While steel prices are clearly exogenous to both the wind and solar industries, silicon prices are more of a gray area, straddling the border between being an exogenous or endogenous influence on solar’s CapEx. Clearly, the solar industry has played a role in past periods of silicon price volatility (Nemet, 2019)—potentially arguing for endogenous treatment. Yet, on the other hand, the solar industry reportedly accounts for only about 20% of total demand for silicon, and is only the third-largest source of demand (Desai, 2021), after aluminum alloys (∼45%) and silicones (∼30%)—potentially arguing for exogenous treatment. This uncertainty over the endogeneity of silicon prices renders both options—i.e., either including it as a normalization factor (as we have) or not—as not fully satisfactory. Figure S4 of the supplemental information shows that if we were to instead not normalize solar’s CapEx using silicon prices, then solar’s full-period learning rate would increase from 23.6% to 25.3% (reflecting the fact that our utility-scale PV LCOE time series begins in 2007, which was a time of high silicon prices).

We control for fluctuations in exchange rates over time by adopting the approach taken by Bolinger and Wiser (2012), which applies temporal changes in a trade-weighted basket of exchange rates (representing the primary exporters of wind turbines and PV modules to the U.S.) to the proportion of plant capital costs subject to exchange rate risk. Trade weights are based on a review of International Trade Commission data for wind (Wiser et al., 2021a) and the EIA’s PV module shipment reports for solar (https://www.eia.gov/renewable/monthly/solar_photo/), and annual changes to the resulting currency basket over time are indexed to 2020. To estimate the amount of CapEx subject to exchange rate risk, we multiply the proportion of total plant CapEx attributable to wind turbines and PV modules (i.e., the two most likely components to be imported) by the overall fraction of those components that have been imported over time (from same sources as above). We then reduce the resulting product by half to reflect incomplete exchange rate pass-through (Bolinger and Wiser, 2012). For wind, the resulting exchange rate exposure ranges from approximately 15%–25% depending on the year; for solar, it varies more tightly around a 15% level over time. Finally, we multiply each plant’s exchange rate exposure by the indexed currency basket in the appropriate COD year to assess the impact of exchange rather movements on plant CapEx, relative to the 2020 base year.

We control for the impact of tariffs on capital costs (solar only) by comparing U.S. and global average selling prices for PV modules (Feldman et al., 2021; Wood, 2020/SEIA, 2021; Barbose et al., 2020), and assuming that the difference between them (i.e., the U.S. price premium) is attributable to U.S. tariffs on PV modules, steel, and other commodities. Once again, we index changes in the delta over time to 2020.

Egli et al. (2018) find evidence of learning with respect to renewable energy project finance, yet financing costs are also clearly subject to macroeconomic forces beyond the control of wind and solar developers. We control for these macroeconomic influences by giving credit only to improvements in wind and solar financing costs that exceed those experienced by the broader economy. As noted earlier, wind- and solar-specific financing costs are based on representative interest rates and expected equity returns (both expressed on an after-tax basis) that vary yearly, as well as a constant debt/equity ratio of 70%/30% over time for both wind and solar. The broader, economy-wide cost of equity comes from Damodaran (2021), who uses a free cash flow to equity approach to estimate the expected risk premium of (and, when combined with the risk-free Treasury bond rate, the expected total return of) the stock market over time. We combine that expected equity return with the BAA corporate bond yield using the same capital structure of 70% equity/30% debt. Finally, we index the delta between the wind- or solar-specific financing costs and the economy-wide financing cost to 2020, in effect only crediting wind and solar with any financing cost changes that differ from those experienced by the broader economy.

We normalize income tax rates simply by setting them equal to 2020’s values over the entire period.

Finally, for capacity factors, we control for variation in the quality of sites (in terms of wind or solar resource) developed over time to prevent any systematic shifts towards more- or less-energetic sites from masking learning. We accomplish this by multiplying each plant’s levelized capacity factor by the ratio of the generation-weighted average modeled capacity factor across all plants with 2020 CODs (the numerator) to the generation-weighted average modeled capacity factor across all plants with the same COD year as the plant of interest (the denominator). Modeled capacity factors all use the same wind and solar technology assumptions, so that they only vary due to difference in the underlying wind (wind speed) and solar (irradiation) resource. In this way, we normalize all plant-level capacity factors to the average wind or solar resource among plants with 2020 CODs.

Applying these normalization factors as described above results in a normalized LCOE time series that we then use to estimate learning rates.

### Quantification and statistical analysis

#### LCOE learning rate estimation

We tested segmented regression models with the possibilities of no change points (constant learning), a single change point, or multiple change points. To briefly describe segmented regression, recall that standard learning rates can be estimated through a regression of logged price (in our case, logged LCOE) on logged cumulative capacity:

(Equation 2) $$\log\left(LCOE_{t}\right)=\beta_{0}+\beta_{1}\;\log\left(MW_{t}\right)$$

Where $LCOE_{t}$ and $MW_{t}$ are LCOE and cumulative installed MW at time t, respectively, and $\beta_{1}$ is the learning coefficient (which provides the basis for estimating the learning rate). Suppose, for the moment, there is a known change point at time τ. That is, at t=τ there is a significant change in the learning rate. Segmented regression models estimate separate learning rates before and after the change point as follows:

(Equation 3) $$\log\left(LCOE_{t}\right)=\alpha_{0}+\alpha_{1}\;\log\left(MW_{t}\right)+\alpha_{2}\left[\log\left(MW_{t}\right)-\log\left(MW_{\tau }\right)\right]\times I\left(MW_{t}>MW_{\tau }\right)$$

Where $I\left(MW_{t}>MW_{\tau }\right)$ is an indicator variable equal to one for any observations after the change point and equal to 0 for all observations before the change point. The coefficient $\alpha_{2}$ is the difference in the learning coefficient after the change point. Equation (3) can be expanded to include any number of change points.

Muggeo (2008) describes a method for identifying the unknown change point or points. The method involves adding a third term to Equation (3), which can be used to iteratively estimate the change point τ:

(Equation 4) $$\log\left(LCOE_{t}\right)=\alpha_{0}+\alpha_{1}\;\log\left(MW_{t}\right)+\alpha_{2}\left[\log\left(MW_{t}\right)-\log\left(MW_{\tau }\right)\right]\times I^{+}+\gamma I^{-}$$

Where $I^{+}=I\left(MW_{t}>MW_{\tilde{\tau }}\right)$ and $I^{-}=-I\left(MW_{t}>MW_{\tilde{\tau }}\right)$, and $\tilde{\tau }$ is the unknown change point. The additional coefficient γ effectively estimates the differential learning rate before the change point, which should, by definition, be zero. The method iteratively tests different possibilities of the change point $\tilde{\tau }$ and selects the change point that minimizes the additional coefficient (γ≈0), such that the equation collapses back to Equation (3). We implemented the approach using the segmented package in R; see Muggeo (2008).

The method described above identifies separate models for each specified number of change points. That is, the method identifies a model with no change points, a single change point, two change points, and so on. The final step is to select the model with the optimal number of change points. Model selection cannot be based on goodness-of-fit (i.e., R^2^), given that model fitness will always increase with additional coefficients added for additional change points. Instead, consistent with Wei et al. (2017), we selected the model that minimized the Akaike Information Criterion (AIC), a metric that weights improvements in model fitness against added degrees of freedom. The 2-point model minimized AIC in the case of wind, and the 1-point model minimized the AIC in the case of solar. While the segmented regression and AIC model selection process allowed us to rely on purely statistical techniques to identify the change points, it is worth noting that the estimated change points align with readily observed industry trends, as discussed in the Results. See the full segmented model regression results and AIC values in Tables S1 and S2 of the supplemental information.

#### LCOE component learning and impact on LCOE reduction

We explore the learning rates of individual LCOE components, as well as their relative contributions to historical reductions in LCOE. The full-period learning rates for wind and solar shown in Figure 4 are derived by taking the natural logarithm of each individual component (with total CapEx and capacity factor first normalized as described above, but OpEx and Design Life not normalized) and then regressing those logged components against the natural logarithm of cumulative total global wind or solar capacity, as appropriate. The learning rate equals $1-2^{\beta }$, where β is the slope coefficient estimated in Equation (2). Technically, the learning rates for capacity factor and design life are negative (given the increase in each with deployment), but we express them as positive in Figure 4 since, for both components, higher values drive LCOE lower. Figure 5 then assesses the relative contribution of each component to overall LCOE reduction since the start of each market, using the approach described in the text surrounding Figure 5.

#### Future LCOE projections

The LCOE projections in Figure 7 reflect the full-period learning rates from Figure 3 applied to an annual projection of cumulative wind or solar capacity through 2050. The cumulative capacity projections (shown in Figure S8) are simple averages of a number of other forecasts (IEA, 2020; EIA, 2019; DNV, 2020; BloombergNEF, 2020; IRENA, 2020; Wood, 2020), and reflect total (i.e., distributed and utility-scale PV or onshore and offshore wind) global capacity. We explored various other permutations of the learning driver (e.g., onshore-only versus total wind, utility-scale-only versus total solar, U.S.-only versus global capacity, and energy versus capacity), but a total global capacity formulation seemed most defensible (see Tables S9–S12 for more details).

The LCOE projections are based on the forecasting methods described in Lafond et al. (2018). That study develops a method to project point estimates of future costs as well as confidence intervals based on errors that can expand over time. Put another way, the method assumes that forecast errors accumulate over time, such that a projection for 20 years in the future is inherently less certain than a projection for 1 year in the future. Following Lafond et al., we assume that forecasted values follow a normal distribution:

(Equation 5) $${\text{c}}_{t}∼N\left({\hat{c}}_{t},V\left({\hat{c}}_{t}\right)\right)$$

Where ${\text{c}}_{t}$ is the forecasted log LCOE in a given year t, ${\hat{c}}_{t}$ is the mean point estimate of the forecasts, and $V\left({\hat{c}}_{t}\right)$ is the variance of the forecasts. We make the simplifying assumption that forecasting errors are not temporally autocorrelated. Further, we assume that historic deployment rates have been relatively constant, a generally defensible assumption for exponential growth (Lafond et al., 2018). Under these assumptions, Lafond et al. show that the two parameters in Equation 5 can be approximated through the following equations:

(Equation 6) $${\hat{\text{c}}}_{t}=c_{2020}+\beta \left(d_{t}-d_{2020}\right)$$

(Equation 7) $$V\left({\hat{c}}_{t}\right)=\sigma^{2}\left(t+\frac{t^{2}}{T-1}\right)$$

Where β is the coefficient estimated in Equation (2), $d_{t}$ is cumulative (logged) deployment at time t, $\sigma^{2}$ is the variance of the learning rate regression coefficient from Equation (2), and T is the total number of time periods in the time series.

One consequence of accumulating forecast error is that upper-bound LCOE projections can exceed current LCOE. While the pace and magnitude of future LCOE reductions is uncertain, it is highly unlikely that wind and solar LCOE will increase over the long term, especially after normalizing for exogenous factors. We use a bootstrapping approach to provide more realistic upper bounds for our forecast errors. We project 1 million random estimates of future LCOE in every year using the distribution described in Equation (5). We then assume that the absolute maximum of the true distribution is given by LCOE in 2020 and drop projected LCOEs that exceed that value. We then estimate the 90^th^ percentile of the absolute values of all the projected LCOEs in these restricted distributions and use that value as the upper bound of our forecasted LCOE confidence intervals. The lower bounds are based on the 95% confidence intervals constructed from Equation (7). In the case of accelerated learning, we use the regression coefficient $\alpha_{2}$ and standard error from Equation (4).

## Data Availability

• All data reported in this paper are available in the supplemental information. • All original code used in this paper is available in the supplemental information. • Any additional information required to reanalyze the data reported in this paper is available from the lead contact upon request.

## Supplemental information

Document S1. Figures S1–S8 and Tables S1–S16

Data S1. Data and Scripts, related to Figures 1–7, STAR Methods, and Document S1 Data factor_delta_data: Data related to Figure 5 lcoe_learning_data: Annual datasets with inputs to all learning calculations, includes separate files for solar and wind (related to Figures 1–4, 6, and 7) project_level: Project-level LCOE estimates (related to Figure 1) SI Data: Additional data files related to Figure S1, S2, and S4; Tables S1–S4 and Tables S13–S16 Scripts figures_1_2_4_5: Scripts to generate Figures 1, 2, 4, and 5 forecasts: Script to generate LCOE-learning based forecasts (related to Figures 6 and 7) learning_curves: Scripts to run segmented regression models (related to Figure 3) prep: A base script that is run to load all data (this is run automatically within the other scripts) SI Scripts: Additional scripts related to Figures S1, S2, and S4; Tables S1–S4 and Tables S13–S16.
