# Supplementary material for: Levelized cost-based learning analysis of utility-scale wind and solar in the United States
Source: iScience. 2022 May 9;25(6):104378. doi: 10.1016/j.isci.2022.104378 (PMC9127581; doi:10.1016/j.isci.2022.104378)
Supplement: Document S1. Figures S1–S8 and Tables S1–S16 [file mmc1.pdf]

iScience, Volume *25*

Supplemental information

Levelized cost-based learning analysis of utility-scale wind and solar in the United States

Mark Bolinger, Ryan Wiser, and Eric O'Shaughnessy

	No CP	1 CP	2 CP
**Epoch 1**	-0.23* (0.01)	-0.2* (0.01)	-0.25* (0.02)
**Epoch 2**		-0.49 (0.3)	0.38 (0.2)
**Epoch 3**			-0.87* (0.24)
**AIC**	-74.57	-80.27	-87.4
**R^2**	0.90	0.92	0.94

* p<0.05

Table S1. Segmented Regression Results for Wind

This table is related to Figure 3. It shows the complete numerical results for the segmented regressions for wind, comparing models with no change points (CPs), a single CP, and two CPs. The values are regression coefficients with standard errors in parentheses. The AIC is the Akaike Information Criterion used for model selection (the greater the absolute value of the AIC, the better the model). To ease comparisons with our preferred specifications, throughout this SI we highlight the core “No CP” model results in blue and the core segmented regression results in orange.

	No CP	1 CP
**Epoch 1**	-0.39* (0.05)	-0.18* (0.06)
**Epoch 2**		-0.67* (0.14)
**AIC**	-16.22	-29.46
**R^2**	0.80	0.93

* p<0.05

Table S2. Segmented Regression Results for Solar

This table is related to Figure 3. It shows the complete numerical results for the segmented regressions for solar, comparing models with no change points (CPs) and a single CP. The values are regression coefficients with standard errors in parentheses. The AIC is the Akaike Information Criterion used for model selection (the greater the absolute value of the AIC, the better the model). To ease comparisons with our preferred specifications, throughout this SI we highlight the core “No CP” model results in blue and the core segmented regression results in orange.

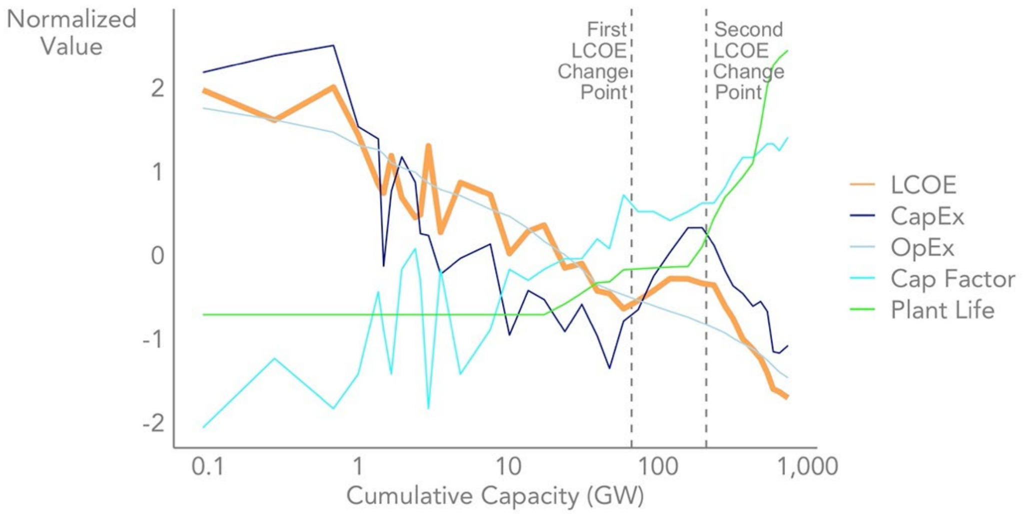

Figure S1. Normalized temporal trends for wind LCOE and cost and plant metrics

This figure is related to Figures 3-5. To visually compare trends in LCOE and its components, we normalize the values such that each metric has a mean value of zero. The two vertical dashed lines correspond to the change points identified for wind LCOE. A visual inspection suggests that CapEx followed a similar trajectory as overall LCOE, with a temporary increase in CapEx occurring around the first LCOE change point and a subsequent decline in CapEx coinciding with the second change point. There is also a noticeable upward break in plant lifetime occurring near the second change point. Capacity factors tended to move upward prior to the first and after the second LCOE change point but were stable during the period of rising LCOE between those two change points. This brief period of capacity factor stability in between the two change points coincides with a period of stagnation in both hub heights and rotor diameters—both around 80 meters, on average—as wind turbine manufacturers grappled with size-related transportation constraints while simultaneously attempting to contain the impact of rising CapEx over this same period.

Change Point		LCOE	CapEx	OpEx	Capacity Factor	Plant Life
1	MW	66,818	46,348	701	2,382	13,139
	Year	2006	2004	1984	1991	1999
2	MW	210,591	167,538	538,198	4,410	217,603
	Year	2010	2009	2017	1995	2011

Table S3. Change Points for Wind LCOE and Individual Components

This table is related to Figures 3-5. Here and throughout this Supplemental Information, we provide estimated change points in terms of cumulative installed capacity (MW) at the change point and the year in which the change point occurs. Consistent with Figure S1, the two CapEx change points align most closely with the two LCOE change points, suggesting that changes in wind LCOE around these change points tracked most closely with changes in CapEx. The second change point for plant life coincides very closely with the second change point in LCOE. Collectively, these results suggest that the first LCOE change point (reversed learning) can mostly be attributed to increasing CapEx over that time period, while the second change point (accelerated learning) can be attributed in significant measure to a combination of falling CapEx and increasing plant life.

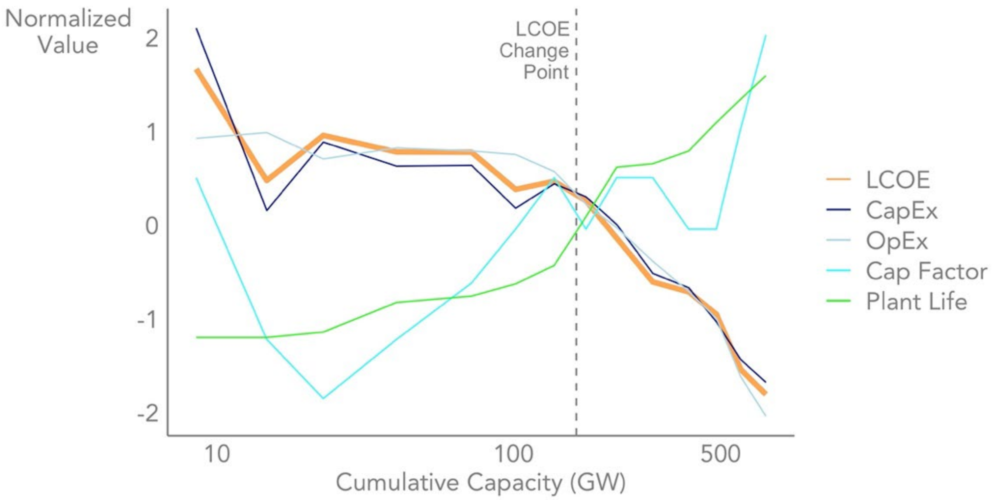

Figure S2. Normalized temporal trends for solar LCOE and cost and project metrics
This figure is related to Figures 3-5. The vertical dashed line corresponds to the change point identified for solar LCOE. A visual inspection suggests that solar LCOE closely tracks with CapEx, and that the accelerated learning that occurs after the change point is partly attributable to accelerating reductions in CapEx and OpEx that occurred around the same time, as well as improvements in plant life.

Change Point		LCOE	CapEx	OpEx	Capacity Factor	Plant Life
1	MW	162,785	205,095	146,657	17,690	90,384
	Year	2014	2015	2013	2008	2012

Table S4. Change Points for Solar LCOE and Individual Components
This table is related to Figures 3-5. Consistent with Figure S2, the LCOE change point coincides closely with the change point in both CapEx and OpEx.

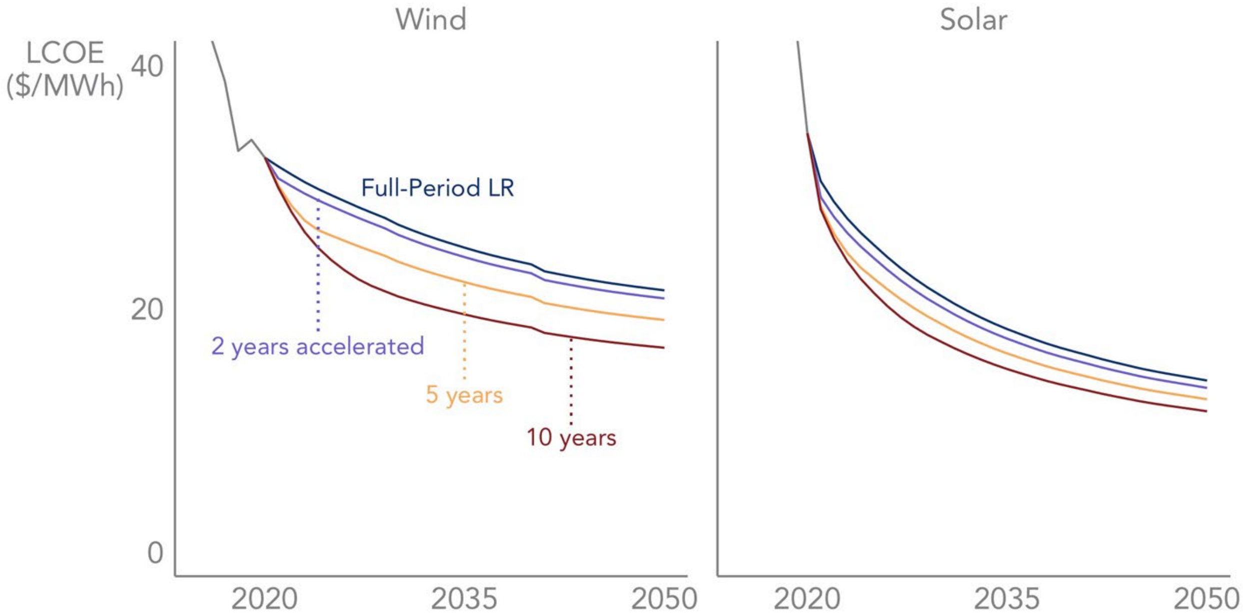

Figure S3. LCOE projection ranges based on full-period and accelerated learning rates

This figure is related to Figures 3 and 7. It illustrates the possibility that accelerated learning in recent years represents only a temporary phenomenon, by plotting LCOE projections based on different assumptions about how quickly wind and solar learning rates return to background (full-period) learning rates. Projections based on accelerated learning rates assume that learning converges on the full-period learning rate within 2 years (purple), 5 years (orange), or 10 years (red). The longer that the accelerated learning is sustained, the lower the long-term LCOE, all else equal. Moreover, the relative long-term impacts of accelerated learning depend on how much faster is the accelerated learning rate than the background learning rate. In this case, the accelerated learning rates are about 2.7 and 1.9 times faster than the background learning rates for wind and solar, respectively. As a result, the remaining duration of accelerated learning has a greater long-term impact on projected wind LCOE than on solar LCOE.

CP		Normalized	Raw
1	MW	66,818	53,046
	Year	2006	2004
2	MW	210,591	187,724
	Year	2010	2010

Table S5. Wind Change Points with Normalized vs. Raw LCOE

This table is related to Figures 1 and 2 and STAR Methods.

	No CP		2 CP	
	Normalized	Raw	Normalized	Raw
LR1	15%*	18%*	16%*	21%*
LR2			-10%	-23%*
LR3			40%*	46%*
AIC	-74.57	-53.26	-87.4	-71.56
R^2	0.90	0.89	0.94	0.94

* p<0.05

Table S6. Wind Learning Rates with Normalized vs. Raw LCOE

This table is related to Figures 1 and 2 and STAR Methods.

CP		Normalized	Raw
1	MW	162,785	159,006
	Year	2014	2014

Table S7. Solar Change Points with Normalized vs. Raw LCOE

This table is related to Figures 1 and 2 and STAR Methods.

	No CP		1 CP	
	Normalized	Raw	Normalized	Raw
LR1	24%*	27%*	12%*	17%*
LR2			45%*	45%*
AIC	-16.22	-19.5	-29.46	-32.98
R^2	0.80	0.96	0.93	0.96

* p<0.05

Table S8. Solar Learning Rates with Normalized vs. Raw LCOE

This table is related to Figures 1 and 2 and STAR Methods.

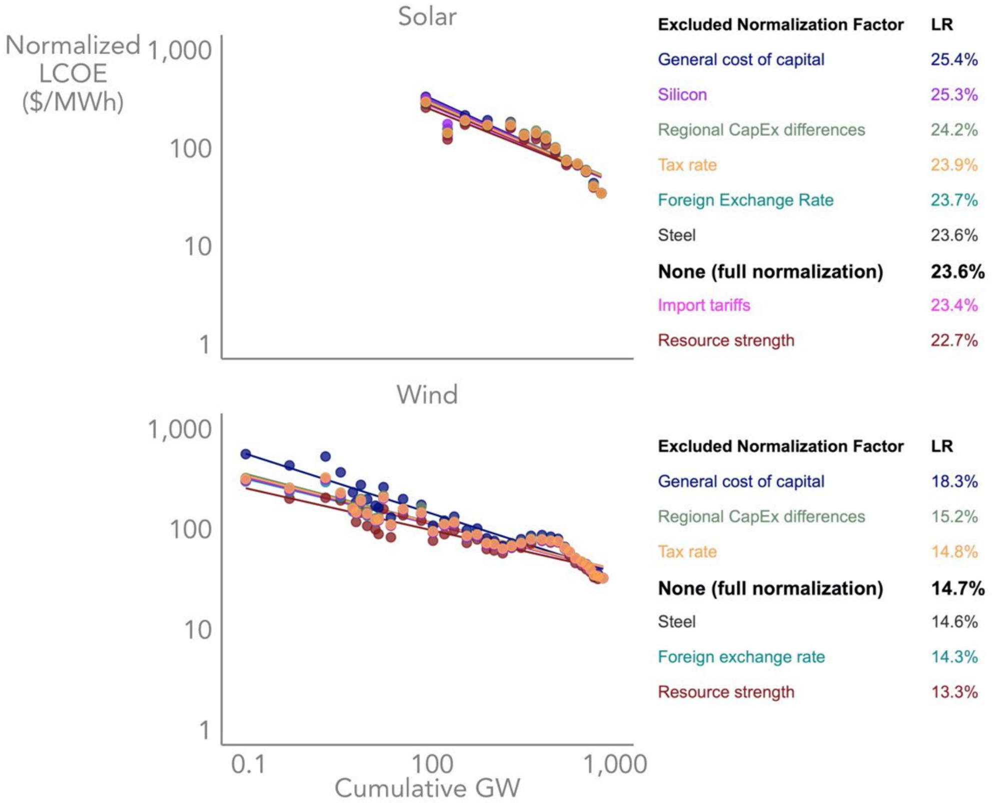

Figure S4. Learning curves based on LCOE when excluding specific normalization factors
This figure is related to Table 1, Figures 2 and 3, and STAR Methods. It illustrates estimated LCOE and learning rates when excluding individual normalization factors. For instance, in the first row of the plot, the dark blue points depict solar LCOE when normalizing for all factors except cost of capital, which yields a learning rate of 25.4%. The estimated LCOE and learning rates are generally similar but the figure suggests that normalized LCOE and learning rates are particularly sensitive to a few key factors, especially general cost of capital, silicon (in the case of solar), regional CapEx differences, and resource strength. For all other normalization factors (e.g., foreign exchange rates, tax rate), the learning rate is relatively insensitive as to whether or not such factors are normalized.

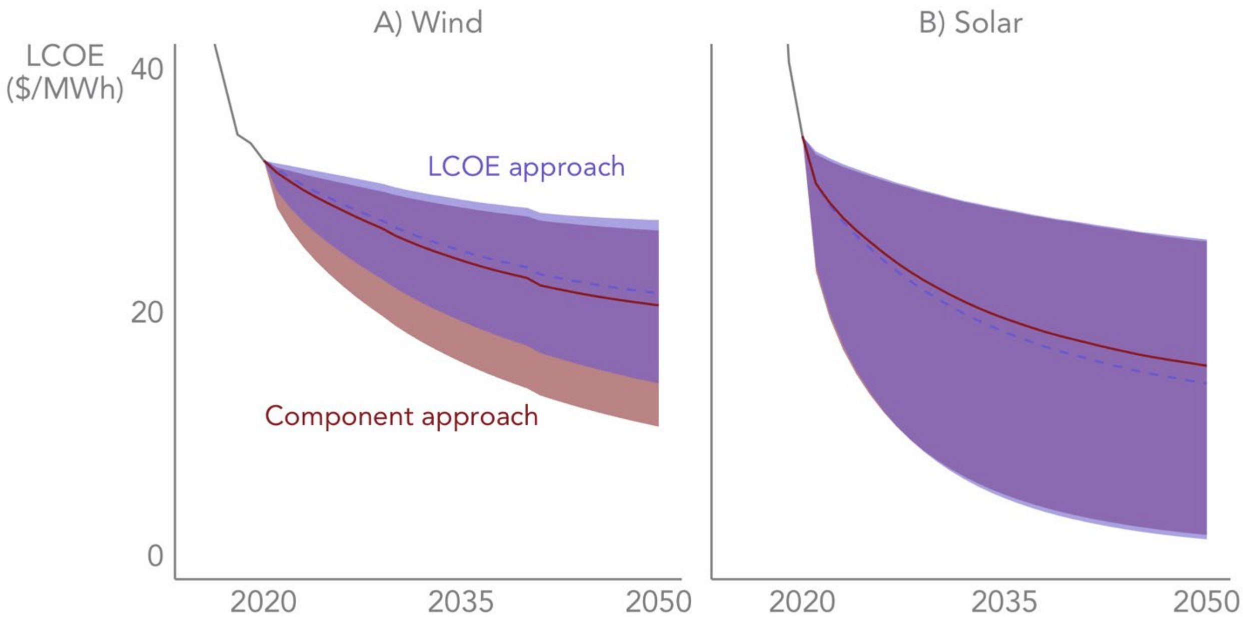

Figure S5. Comparison of LCOE projections using the LCOE and component approaches
This figure is related to Figure 7. LCOE projections can be constructed in at least two ways: based on temporal covariation between LCOE and cumulative installed capacity (the "LCOE approach"), or by estimating learning rates for the individual component inputs to LCOE, and then calculating LCOE based on the combined projections of those components (the "component approach"). We used the LCOE approach, while accounting for exogenous factors through normalization. This figure shows that our results and discussion would be largely the same had we instead used the component approach.

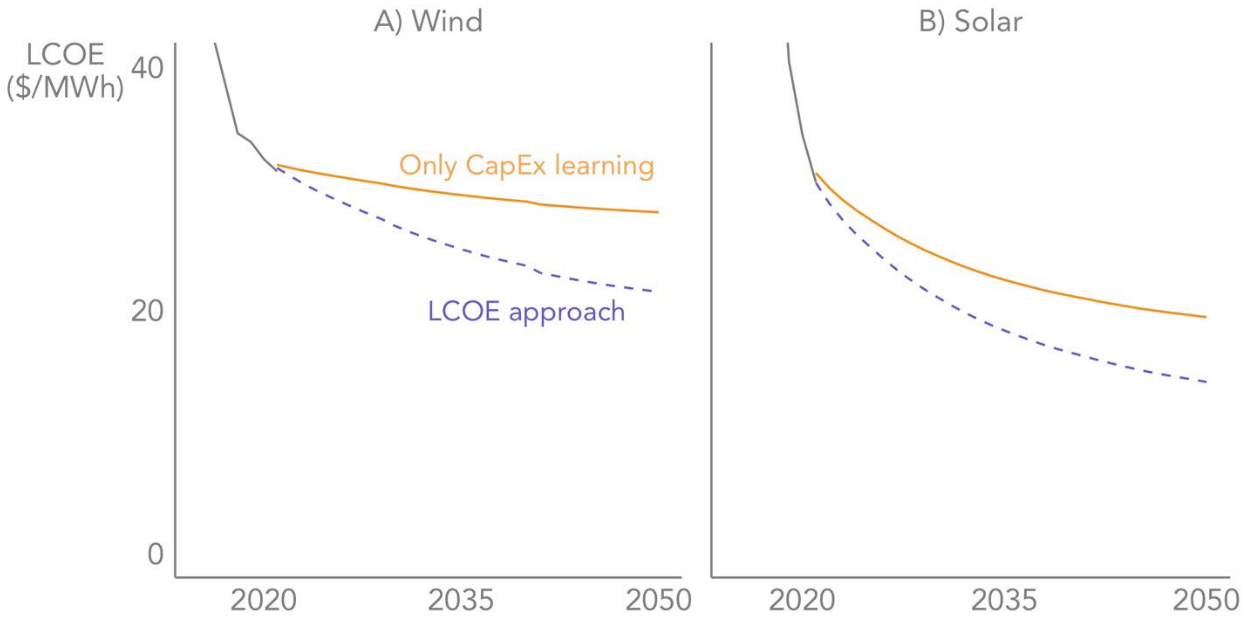

Figure S6. Projected LCOEs assuming only CapEx learning (orange solid lines) versus learning in all four LCOE components (blue dashed lines)
This figure is related to Figures 4 and 7. It demonstrates that a CapEx-based learning rate tells only a partial story of overall LCOE reduction in wind and solar. The "Only CapEx learning" line (solid orange) projects future LCOE based on learning-based projections for CapEx but holding all other LCOE components constant. The gaps between the two lines represent projected LCOE reductions that are attributable to declining OpEx and increasing capacity factors and plant lifetimes.

CP		Global MW (Core)	Global Onshore MW	U.S. MW	U.S. Onshore MW	Global GWh	Global Onshore GWh	U.S. GWh	U.S. Onshore GWh
1	**MW**	66,818	66,488	10,044	10,044	671,511	660,487	1,044	1,044
	**Year**	2006	2006	2005	2005	2006	2006	1984	1984
2	**MW**	210,591	208,639	43,506	43,510	1,522,234	1,479,257	834,242	834,379
	**Year**	2010	2010	2010	2010	2010	2010	2013	2013

Table S9. Sensitivities in Wind Change Points

This table is related to Figure 3 and STAR Methods. It presents change point results for eight variations of wind output. The MW definitions all yield comparable change points to the core MW definition (shaded in orange). The global GWh definitions identify similar change points as the core MW model, while the U.S. GWh definitions identify significantly earlier initial change points.

	Global MW (Core)	Global Onshore MW	U.S. MW	U.S. Onshore MW	Global GWh	Global Onshore GWh	U.S. GWh	U.S. Onshore GWh
LR1	16%*	16%*	23%*	23%*	13%*	13%*	5%	5%
LR2	-10%	-10%	-8%*	-8%*	-13%	-13%	15%	15%
LR3	40%*	41%*	48%*	48%*	28%	28%	28%	28%
AIC	-87.4	-87.37	-75.97	-75.97	-84.78	-84.59	-73.8	-73.8
R^2	0.94	0.94	0.91	0.91	0.93	0.93	0.91	0.91

* p<0.05

Table S10. Sensitivities in Wind Learning Rates

This table is related to Figure 3 and STAR Methods. It presents the learning rate results for the eight variations of wind output based on the 2-change point model. Like the change point results, the learning rate results are generally similar across the MW definitions but inconsistent across the GWh definitions. The core model (shaded in orange) has the highest R^2 and AIC.

CP		Global MW (Core)	Global Utility-Scale MW	U.S. MW	U.S. Utility-Scale MW	Global GWh	Global Utility-Scale GWh	U.S. GWh	U.S. Utility-Scale GWh
1	**MW**	162,785	110,578	22,220	11,780	572,470	294,916	77,676	35,360
	**Year**	2014	2014	2014	2014	2014	2014	2014	2014

Table S11. Sensitivities in Solar Change Points

This table is related to Figure 3 and STAR Methods. It presents change point results for eight variations of solar output. All eight definitions yield comparable change points to the core MW definition (in orange).

	Global MW (Core)	Global Utility-Scale MW	U.S. MW	U.S. Utility-Scale MW	Global GWh	Global Utility-Scale GWh	U.S. GWh	U.S. Utility-Scale GWh
LR1	12%*	10%*	9%*	6%*	12%*	8%*	12%*	6%*
LR2	45%*	41%*	43%*	40%*	36%*	32%*	33%*	29%*
AIC	-29.46	-32.46	-27.96	-27.25	-29.3	-31.61	-28.59	-28.66
R^2	0.93	0.94	0.92	0.92	0.93	0.94	0.93	0.93

* p<0.05

Table S12. Sensitivities in Solar Learning Rates

This table is related to Figure 3 and STAR Methods. It presents learning rate results for the eight variations of solar output for the single change point model. The learning rates are generally similar across the alternative definitions. The R^2 and AIC of our core model (in orange) is comparable to, but not the highest among, the alternative specifications; the global, utility-scale model in MW terms has slightly better performance. However, the results in terms of change points and learning rates are broadly similar.

CP		1-Factor	2-Factor
1	MW	66,818	17,401
	Year	2006	2000
2	MW	210,591	52,307
	Year	2010	2004

Table S13. Wind Change Points with 1-Factor vs. 2-Factor Model

This table is related to Figure 3 and STAR Methods. It compares 1-factor learning (our preferred specification, shaded in orange) with a 2-factor approach based on lagged cumulative (and depreciating) public R&D expenditures on wind (sourced from IEA and covering only IEA countries[1]). The 2-factor model yields very different results, reflecting the significant multicollinearity between cumulative wind capacity and R&D expenditures (the correlation coefficients between these two factors is 0.96).

	No CP		2 CP	
	1-Factor	2-Factor	1-Factor	2-Factor
LR1	15%*	10%	16%*	7%
LR2			-10%	20%*
LR3			40%*	-13%*
AIC	-74.57	-81.68	-87.4	-86.66
R^2	0.90	0.92	0.94	0.94

* $p<0.05$

Table S14. Wind Learning Rates with 1-Factor vs. 2-Factor Model

This table is related to Figure 3 and STAR Methods.

CP		1-Factor	2-Factor
1	MW	162,785	153,650
	Year	2014	2013

Table S15. Solar Change Points with 1-Factor vs. 2-Factor Model

This table is related to Figure 3 and STAR Methods. It compares 1-factor learning (our preferred specification, shaded in orange) with a 2-factor approach based on lagged cumulative (and depreciating) public R&D expenditures on solar.

	No CP		1 CP	
	1-Factor	2-Factor	1-Factor	2-Factor
LR1	24%*	-5%	12%*	15%
LR2			45%*	48%*
AIC	-16.22	-21.19	-29.46	-27.56
R^2	0.80	0.87	0.93	0.92

* $p<0.05$

Table S16. Solar Learning Rates with 1-Factor vs. 2-Factor Model

This table is related to Figure 3 and STAR Methods.

[1] https://www.iea.org/data-and-statistics/data-product/energy-technology-rd-and-d-budget-database-2

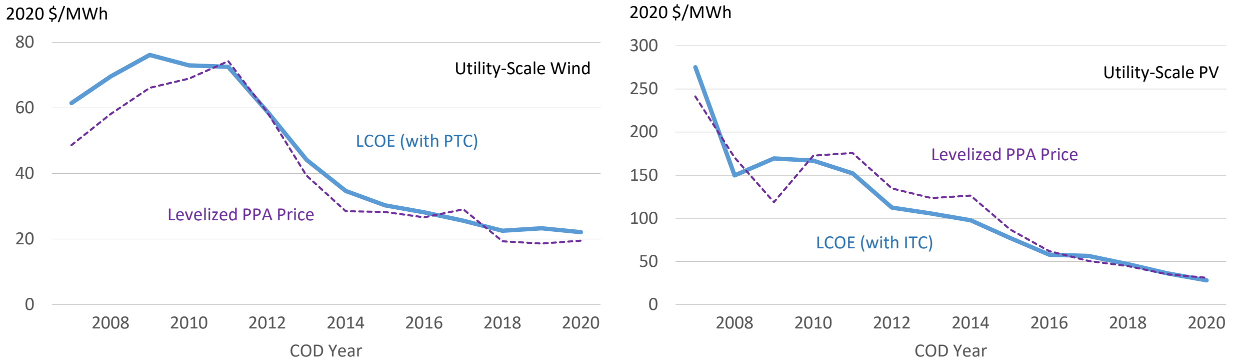

Figure S7. LCOE with tax credits approximates levelized PPA prices

This figure is related to Figure 1. It compares levelized PPA prices to our LCOE time series for utility-scale wind and PV, as adjusted for the PTC and ITC, respectively. The match is not perfect, but the correlation—98% for wind and 95% for PV—is encouraging, and provides confidence in the accuracy of our utility-scale wind and PV LCOE time series.

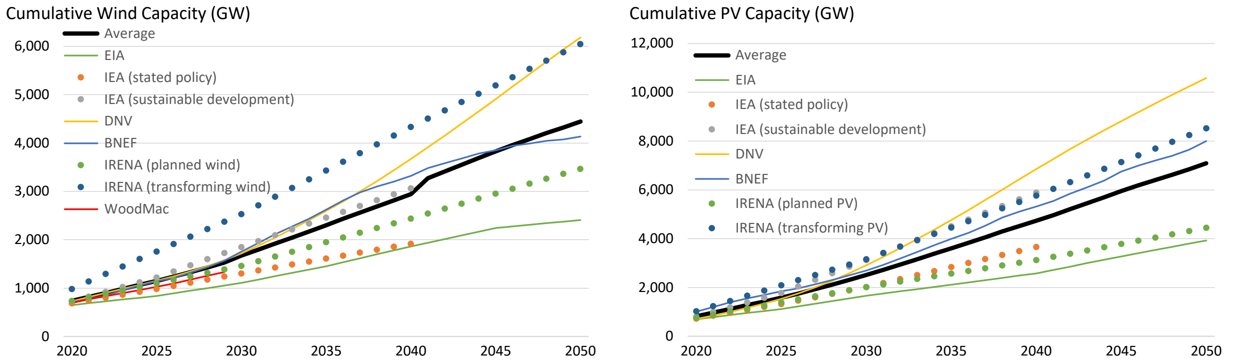

Figure S8. Cumulative global capacity projections for wind and solar

This figure is related to Figures 6 and 7. We use the average projections (solid black lines, which are simple arithmetic means of all other projections shown) as the independent variables to forecast LCOE.
