# Supplementary figures and images for: Levelized cost-based learning analysis of utility-scale wind and solar in the United States
Source: iScience. 2022 May 9;25(6):104378. doi: 10.1016/j.isci.2022.104378 (PMC9127581; doi:10.1016/j.isci.2022.104378)

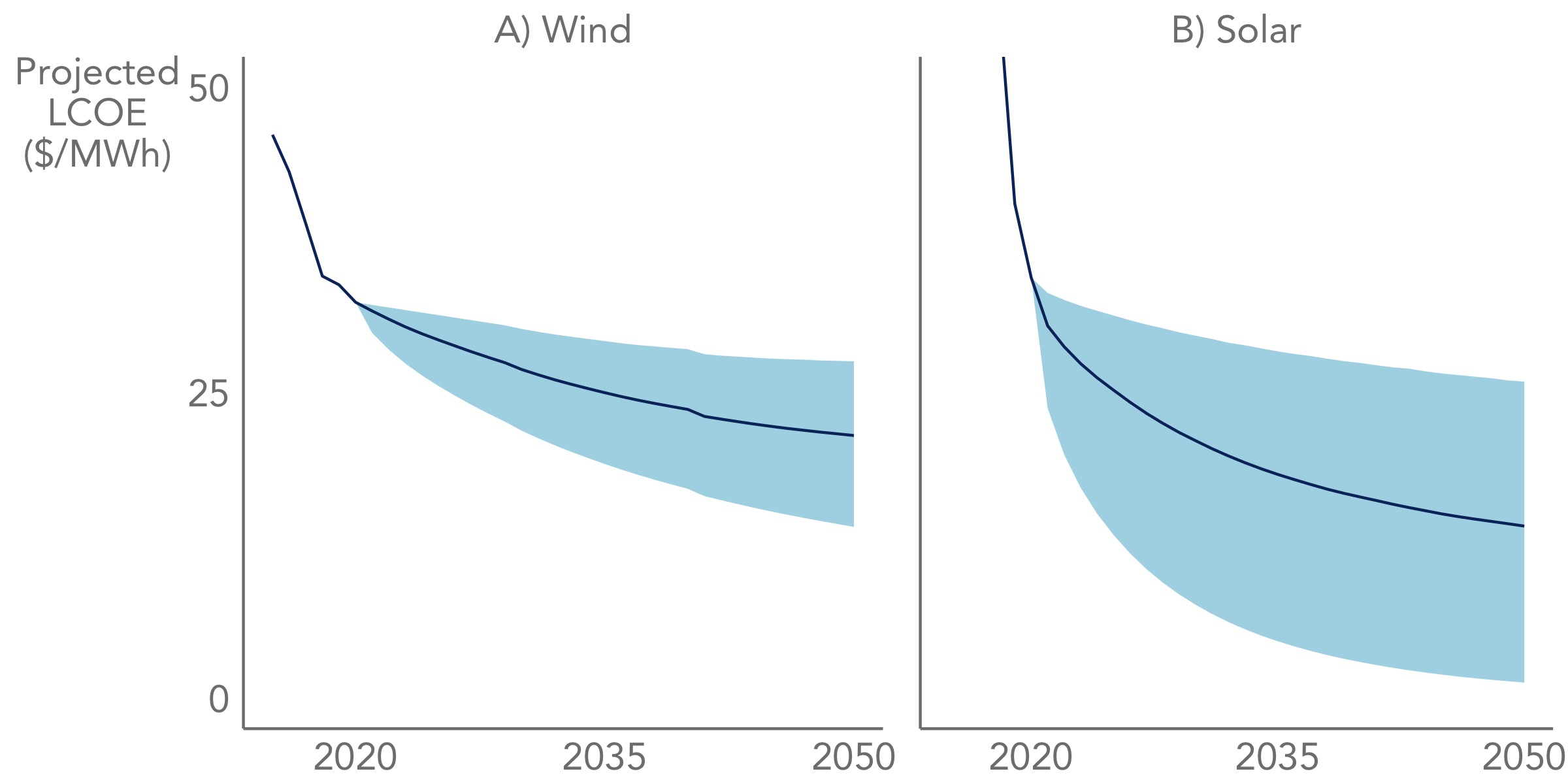

Supplement: Data S1. Data and Scripts, related to Figures 1–7, STAR Methods, and Document S1 — Data factor_delta_data: Data related to Figure 5 lcoe_learning_data: Annual datasets with inputs to all learning calculations, includes separate files for solar and wind (related to Figures 1–4, 6, and 7) project_level: Project-level LCOE estimates (related to Figure 1) SI Data: Additional data files related to Figure S1, S2, and S4; Tables S1–S4 and Tables S13–S16 Scripts figures_1_2_4_5: Scripts to generate Figures 1, 2, 4, and 5 forecasts: Script to generate LCOE-learning based forecasts (related to Figures 6 and 7) learning_curves: Scripts to run segmented regression models (related to Figure 3) prep: A base script that is run to load all data (this is run automatically within the other scripts) SI Scripts: Additional scripts related to Figures S1, S2, and S4; Tables S1–S4 and Tables S13–S16. [file mmc2.zip › Data and Scripts/Figures/f7.jpg]

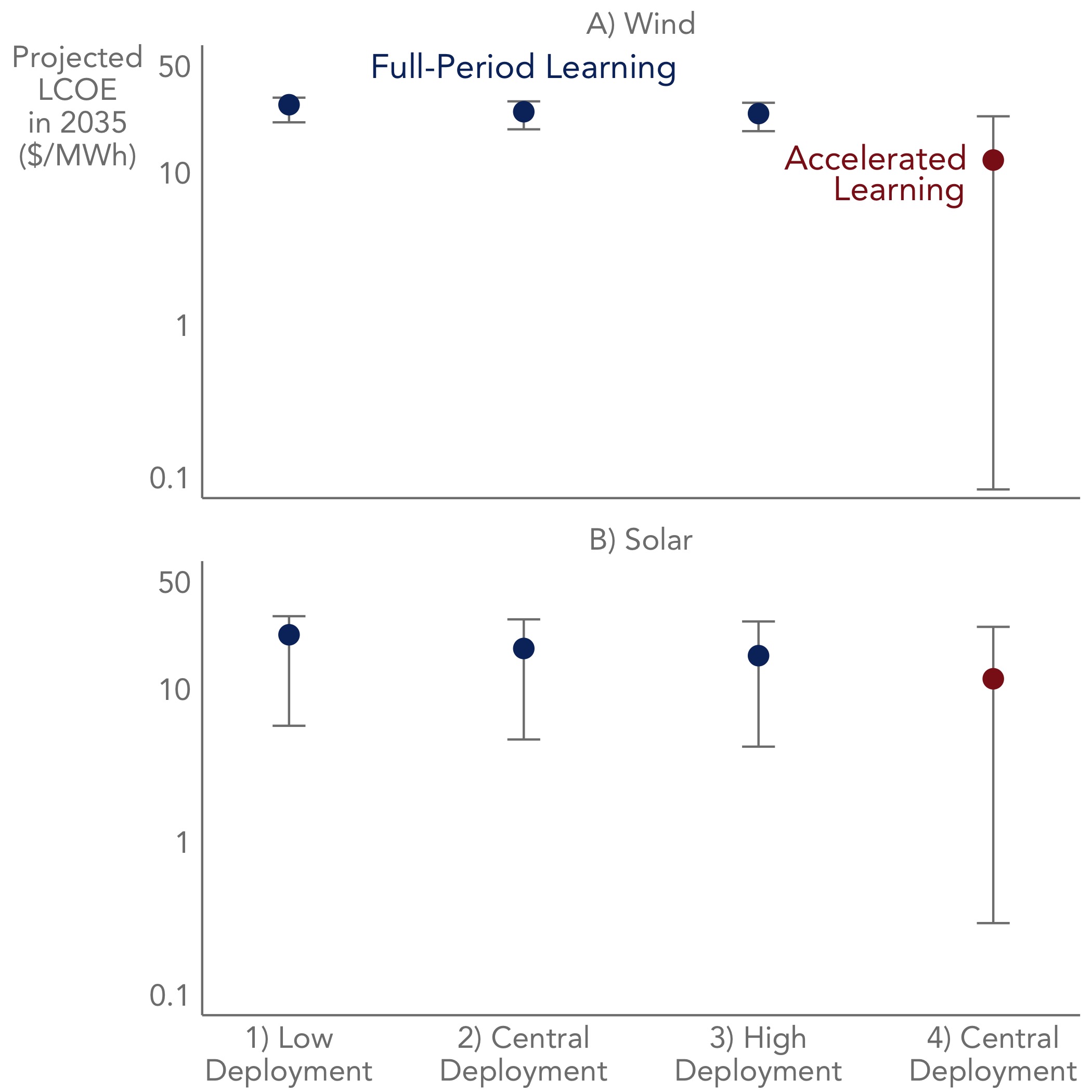

Supplement: Data S1. Data and Scripts, related to Figures 1–7, STAR Methods, and Document S1 — Data factor_delta_data: Data related to Figure 5 lcoe_learning_data: Annual datasets with inputs to all learning calculations, includes separate files for solar and wind (related to Figures 1–4, 6, and 7) project_level: Project-level LCOE estimates (related to Figure 1) SI Data: Additional data files related to Figure S1, S2, and S4; Tables S1–S4 and Tables S13–S16 Scripts figures_1_2_4_5: Scripts to generate Figures 1, 2, 4, and 5 forecasts: Script to generate LCOE-learning based forecasts (related to Figures 6 and 7) learning_curves: Scripts to run segmented regression models (related to Figure 3) prep: A base script that is run to load all data (this is run automatically within the other scripts) SI Scripts: Additional scripts related to Figures S1, S2, and S4; Tables S1–S4 and Tables S13–S16. [file mmc2.zip › Data and Scripts/Figures/f6.jpg]

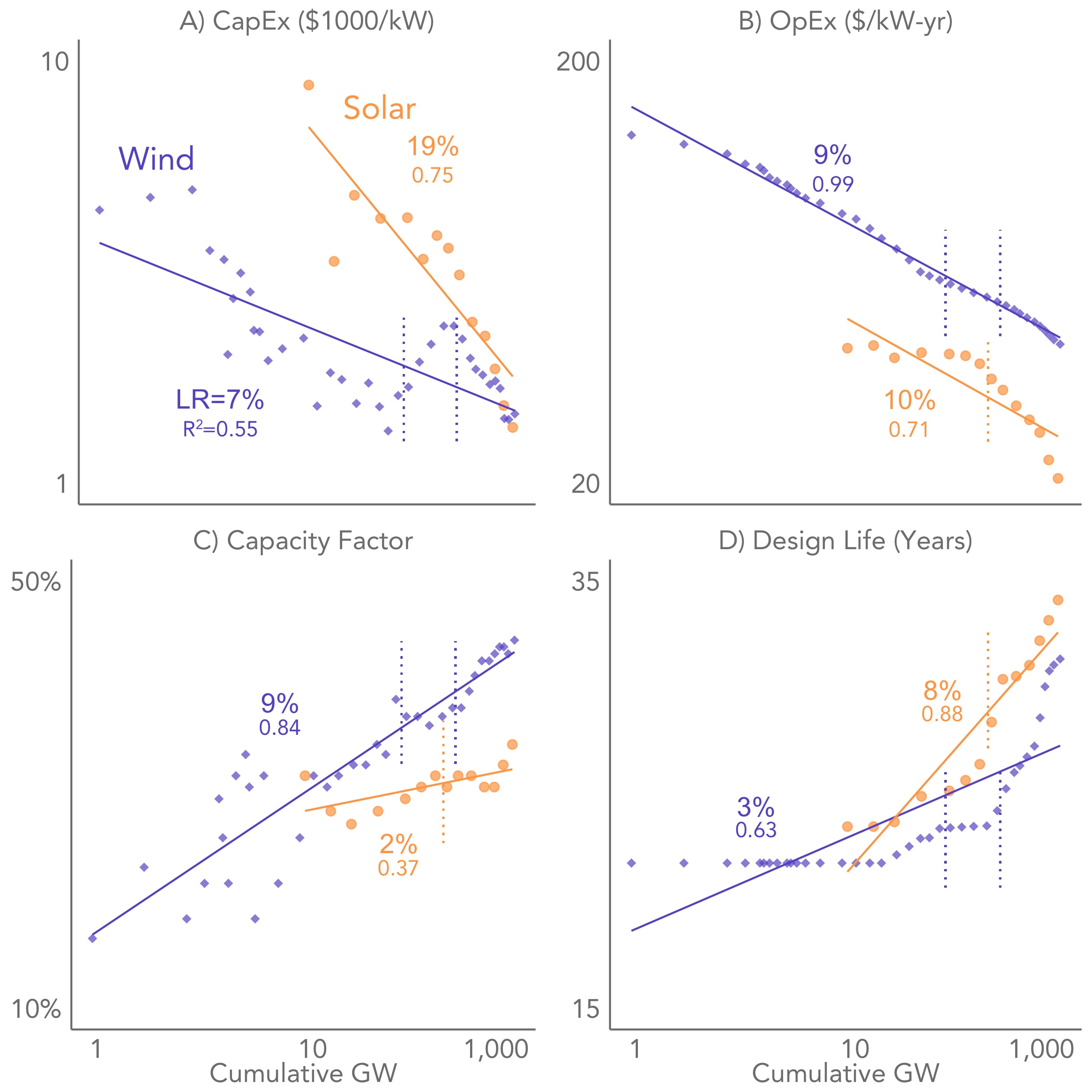

Supplement: Data S1. Data and Scripts, related to Figures 1–7, STAR Methods, and Document S1 — Data factor_delta_data: Data related to Figure 5 lcoe_learning_data: Annual datasets with inputs to all learning calculations, includes separate files for solar and wind (related to Figures 1–4, 6, and 7) project_level: Project-level LCOE estimates (related to Figure 1) SI Data: Additional data files related to Figure S1, S2, and S4; Tables S1–S4 and Tables S13–S16 Scripts figures_1_2_4_5: Scripts to generate Figures 1, 2, 4, and 5 forecasts: Script to generate LCOE-learning based forecasts (related to Figures 6 and 7) learning_curves: Scripts to run segmented regression models (related to Figure 3) prep: A base script that is run to load all data (this is run automatically within the other scripts) SI Scripts: Additional scripts related to Figures S1, S2, and S4; Tables S1–S4 and Tables S13–S16. [file mmc2.zip › Data and Scripts/Figures/f4.jpg]

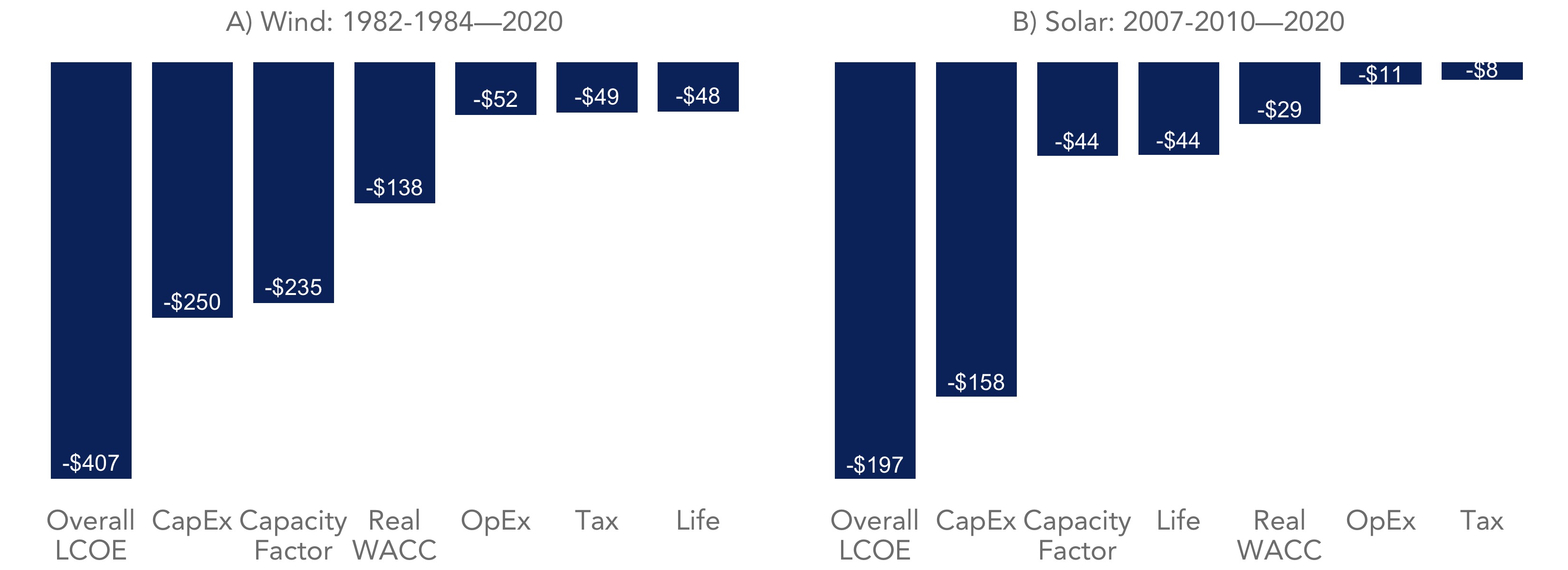

Supplement: Data S1. Data and Scripts, related to Figures 1–7, STAR Methods, and Document S1 — Data factor_delta_data: Data related to Figure 5 lcoe_learning_data: Annual datasets with inputs to all learning calculations, includes separate files for solar and wind (related to Figures 1–4, 6, and 7) project_level: Project-level LCOE estimates (related to Figure 1) SI Data: Additional data files related to Figure S1, S2, and S4; Tables S1–S4 and Tables S13–S16 Scripts figures_1_2_4_5: Scripts to generate Figures 1, 2, 4, and 5 forecasts: Script to generate LCOE-learning based forecasts (related to Figures 6 and 7) learning_curves: Scripts to run segmented regression models (related to Figure 3) prep: A base script that is run to load all data (this is run automatically within the other scripts) SI Scripts: Additional scripts related to Figures S1, S2, and S4; Tables S1–S4 and Tables S13–S16. [file mmc2.zip › Data and Scripts/Figures/f5.jpg]

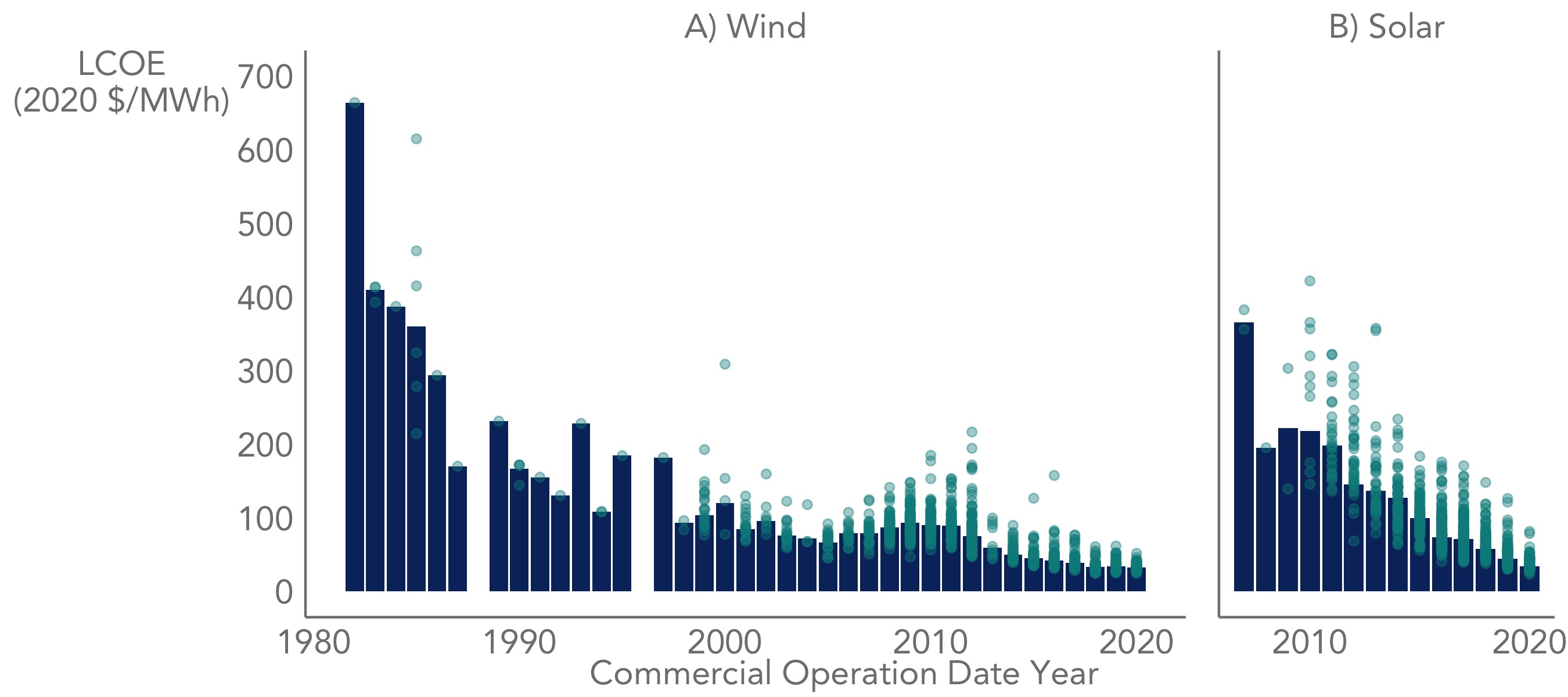

Supplement: Data S1. Data and Scripts, related to Figures 1–7, STAR Methods, and Document S1 — Data factor_delta_data: Data related to Figure 5 lcoe_learning_data: Annual datasets with inputs to all learning calculations, includes separate files for solar and wind (related to Figures 1–4, 6, and 7) project_level: Project-level LCOE estimates (related to Figure 1) SI Data: Additional data files related to Figure S1, S2, and S4; Tables S1–S4 and Tables S13–S16 Scripts figures_1_2_4_5: Scripts to generate Figures 1, 2, 4, and 5 forecasts: Script to generate LCOE-learning based forecasts (related to Figures 6 and 7) learning_curves: Scripts to run segmented regression models (related to Figure 3) prep: A base script that is run to load all data (this is run automatically within the other scripts) SI Scripts: Additional scripts related to Figures S1, S2, and S4; Tables S1–S4 and Tables S13–S16. [file mmc2.zip › Data and Scripts/Figures/f1.jpg]

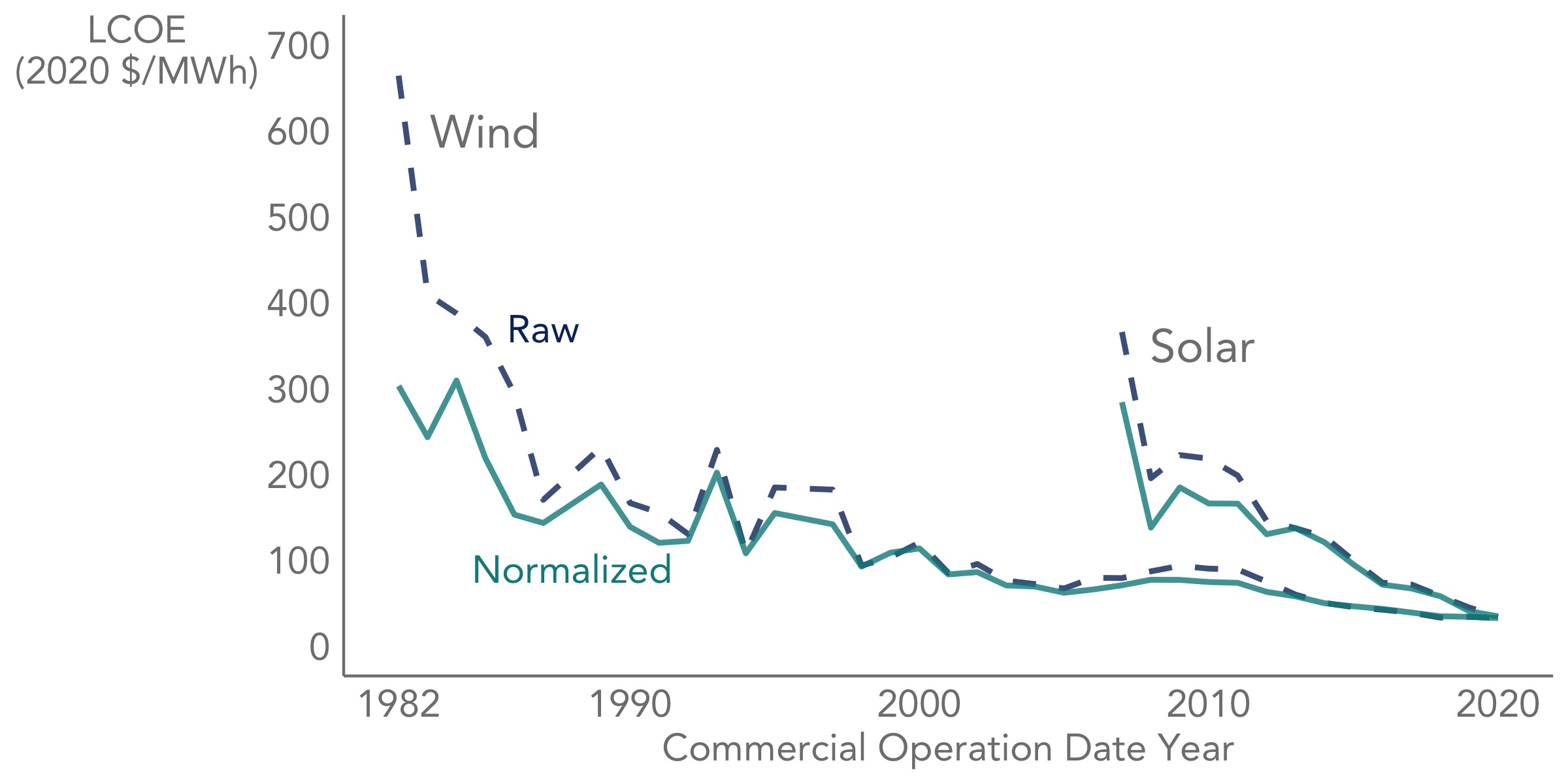

Supplement: Data S1. Data and Scripts, related to Figures 1–7, STAR Methods, and Document S1 — Data factor_delta_data: Data related to Figure 5 lcoe_learning_data: Annual datasets with inputs to all learning calculations, includes separate files for solar and wind (related to Figures 1–4, 6, and 7) project_level: Project-level LCOE estimates (related to Figure 1) SI Data: Additional data files related to Figure S1, S2, and S4; Tables S1–S4 and Tables S13–S16 Scripts figures_1_2_4_5: Scripts to generate Figures 1, 2, 4, and 5 forecasts: Script to generate LCOE-learning based forecasts (related to Figures 6 and 7) learning_curves: Scripts to run segmented regression models (related to Figure 3) prep: A base script that is run to load all data (this is run automatically within the other scripts) SI Scripts: Additional scripts related to Figures S1, S2, and S4; Tables S1–S4 and Tables S13–S16. [file mmc2.zip › Data and Scripts/Figures/f2.jpg]

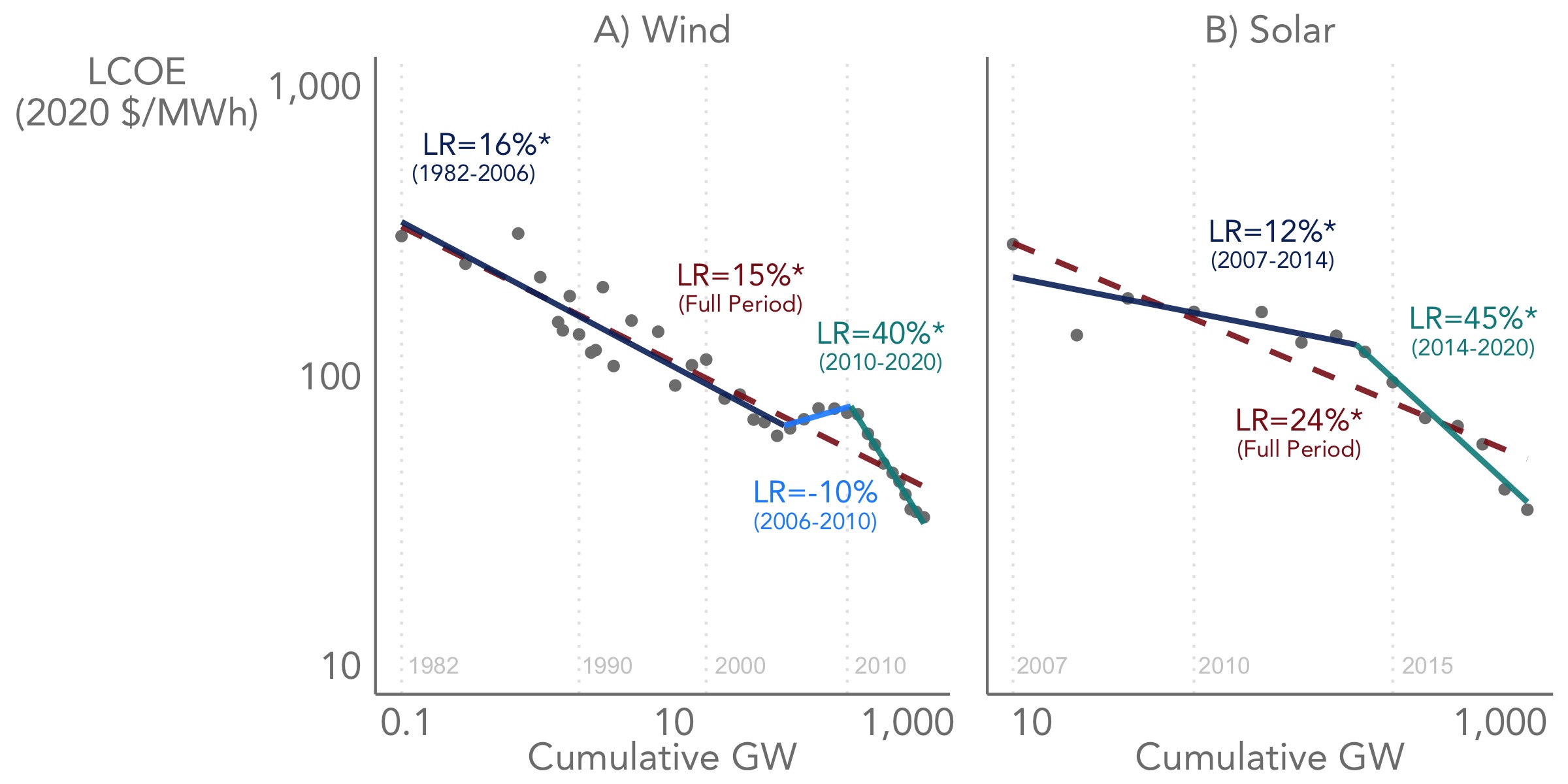

Supplement: Data S1. Data and Scripts, related to Figures 1–7, STAR Methods, and Document S1 — Data factor_delta_data: Data related to Figure 5 lcoe_learning_data: Annual datasets with inputs to all learning calculations, includes separate files for solar and wind (related to Figures 1–4, 6, and 7) project_level: Project-level LCOE estimates (related to Figure 1) SI Data: Additional data files related to Figure S1, S2, and S4; Tables S1–S4 and Tables S13–S16 Scripts figures_1_2_4_5: Scripts to generate Figures 1, 2, 4, and 5 forecasts: Script to generate LCOE-learning based forecasts (related to Figures 6 and 7) learning_curves: Scripts to run segmented regression models (related to Figure 3) prep: A base script that is run to load all data (this is run automatically within the other scripts) SI Scripts: Additional scripts related to Figures S1, S2, and S4; Tables S1–S4 and Tables S13–S16. [file mmc2.zip › Data and Scripts/Figures/f3.jpg]

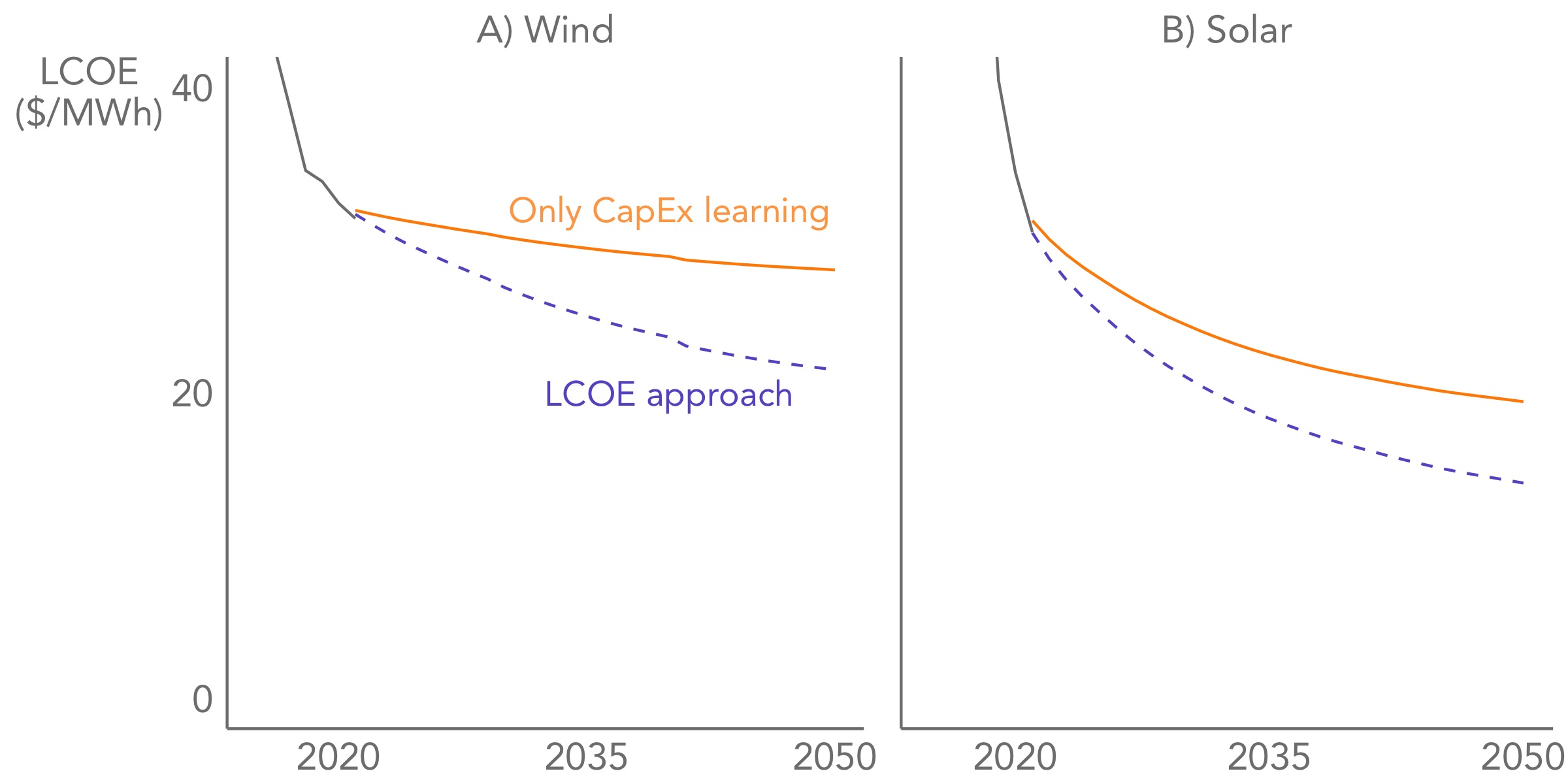

Supplement: Data S1. Data and Scripts, related to Figures 1–7, STAR Methods, and Document S1 — Data factor_delta_data: Data related to Figure 5 lcoe_learning_data: Annual datasets with inputs to all learning calculations, includes separate files for solar and wind (related to Figures 1–4, 6, and 7) project_level: Project-level LCOE estimates (related to Figure 1) SI Data: Additional data files related to Figure S1, S2, and S4; Tables S1–S4 and Tables S13–S16 Scripts figures_1_2_4_5: Scripts to generate Figures 1, 2, 4, and 5 forecasts: Script to generate LCOE-learning based forecasts (related to Figures 6 and 7) learning_curves: Scripts to run segmented regression models (related to Figure 3) prep: A base script that is run to load all data (this is run automatically within the other scripts) SI Scripts: Additional scripts related to Figures S1, S2, and S4; Tables S1–S4 and Tables S13–S16. [file mmc2.zip › Data and Scripts/Figures/SI/only_capex_comparison.jpg]

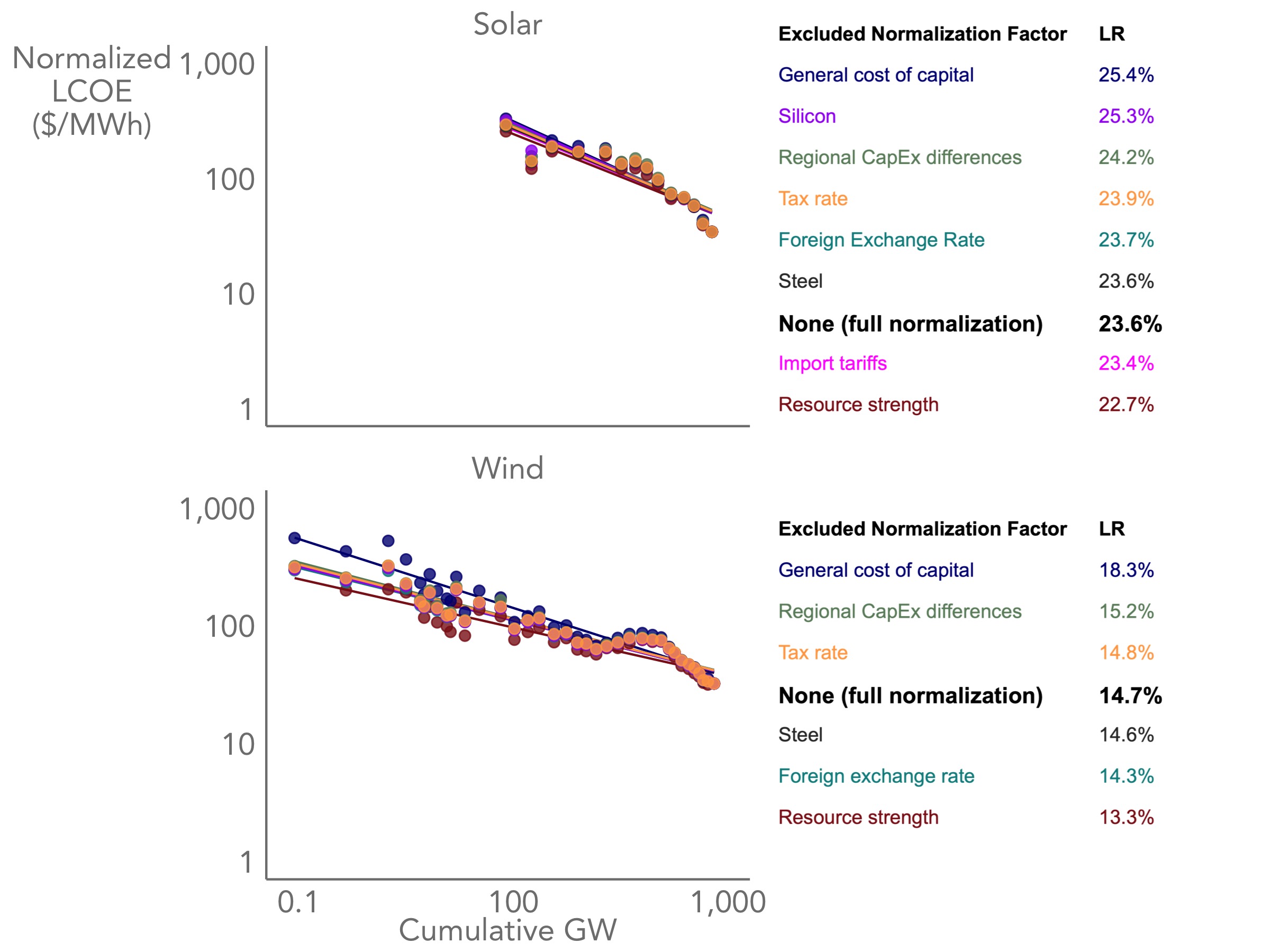

Supplement: Data S1. Data and Scripts, related to Figures 1–7, STAR Methods, and Document S1 — Data factor_delta_data: Data related to Figure 5 lcoe_learning_data: Annual datasets with inputs to all learning calculations, includes separate files for solar and wind (related to Figures 1–4, 6, and 7) project_level: Project-level LCOE estimates (related to Figure 1) SI Data: Additional data files related to Figure S1, S2, and S4; Tables S1–S4 and Tables S13–S16 Scripts figures_1_2_4_5: Scripts to generate Figures 1, 2, 4, and 5 forecasts: Script to generate LCOE-learning based forecasts (related to Figures 6 and 7) learning_curves: Scripts to run segmented regression models (related to Figure 3) prep: A base script that is run to load all data (this is run automatically within the other scripts) SI Scripts: Additional scripts related to Figures S1, S2, and S4; Tables S1–S4 and Tables S13–S16. [file mmc2.zip › Data and Scripts/Figures/SI/normalization_sensitivity.jpg]

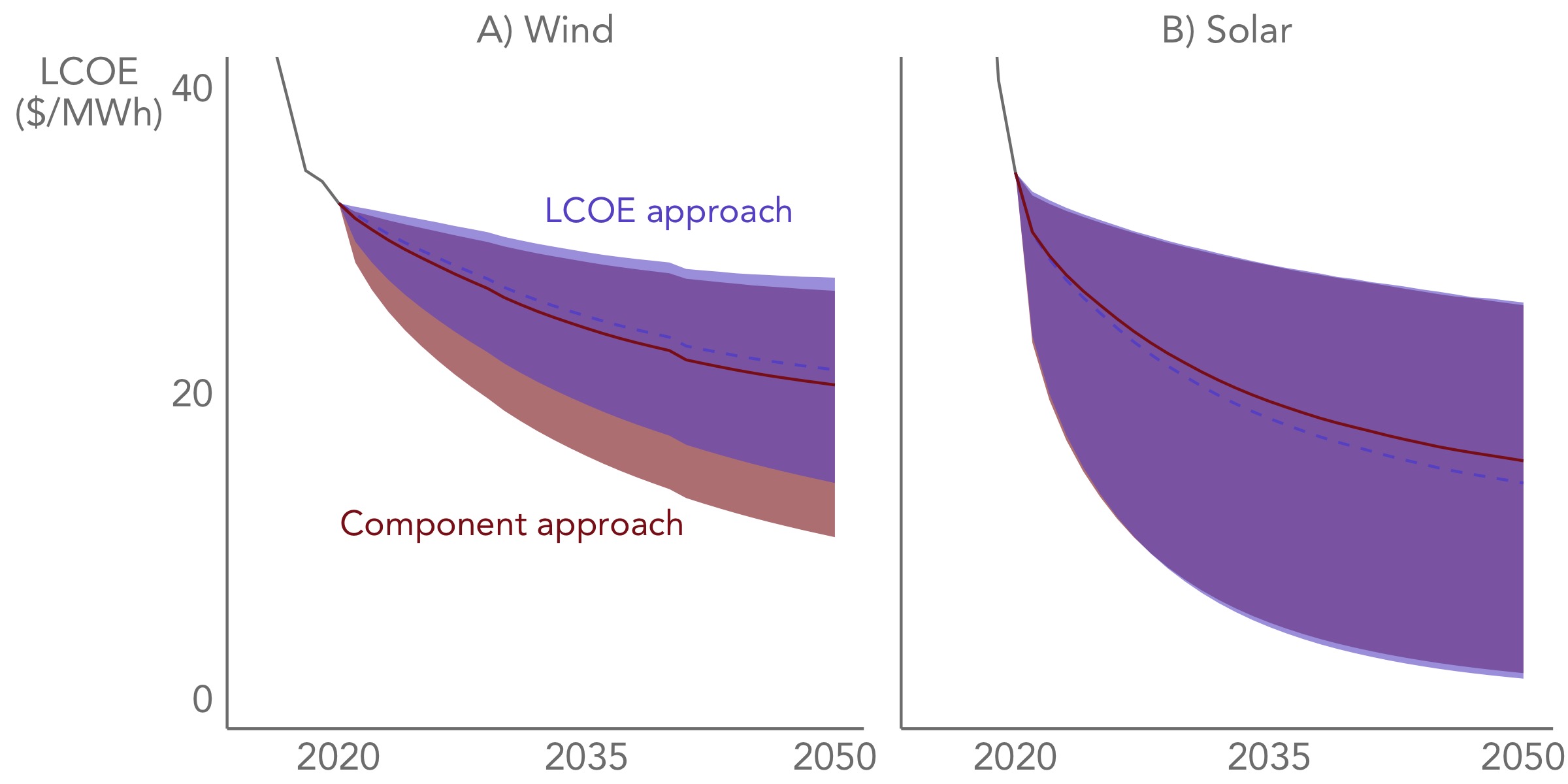

Supplement: Data S1. Data and Scripts, related to Figures 1–7, STAR Methods, and Document S1 — Data factor_delta_data: Data related to Figure 5 lcoe_learning_data: Annual datasets with inputs to all learning calculations, includes separate files for solar and wind (related to Figures 1–4, 6, and 7) project_level: Project-level LCOE estimates (related to Figure 1) SI Data: Additional data files related to Figure S1, S2, and S4; Tables S1–S4 and Tables S13–S16 Scripts figures_1_2_4_5: Scripts to generate Figures 1, 2, 4, and 5 forecasts: Script to generate LCOE-learning based forecasts (related to Figures 6 and 7) learning_curves: Scripts to run segmented regression models (related to Figure 3) prep: A base script that is run to load all data (this is run automatically within the other scripts) SI Scripts: Additional scripts related to Figures S1, S2, and S4; Tables S1–S4 and Tables S13–S16. [file mmc2.zip › Data and Scripts/Figures/SI/component_based_comparison.jpg]

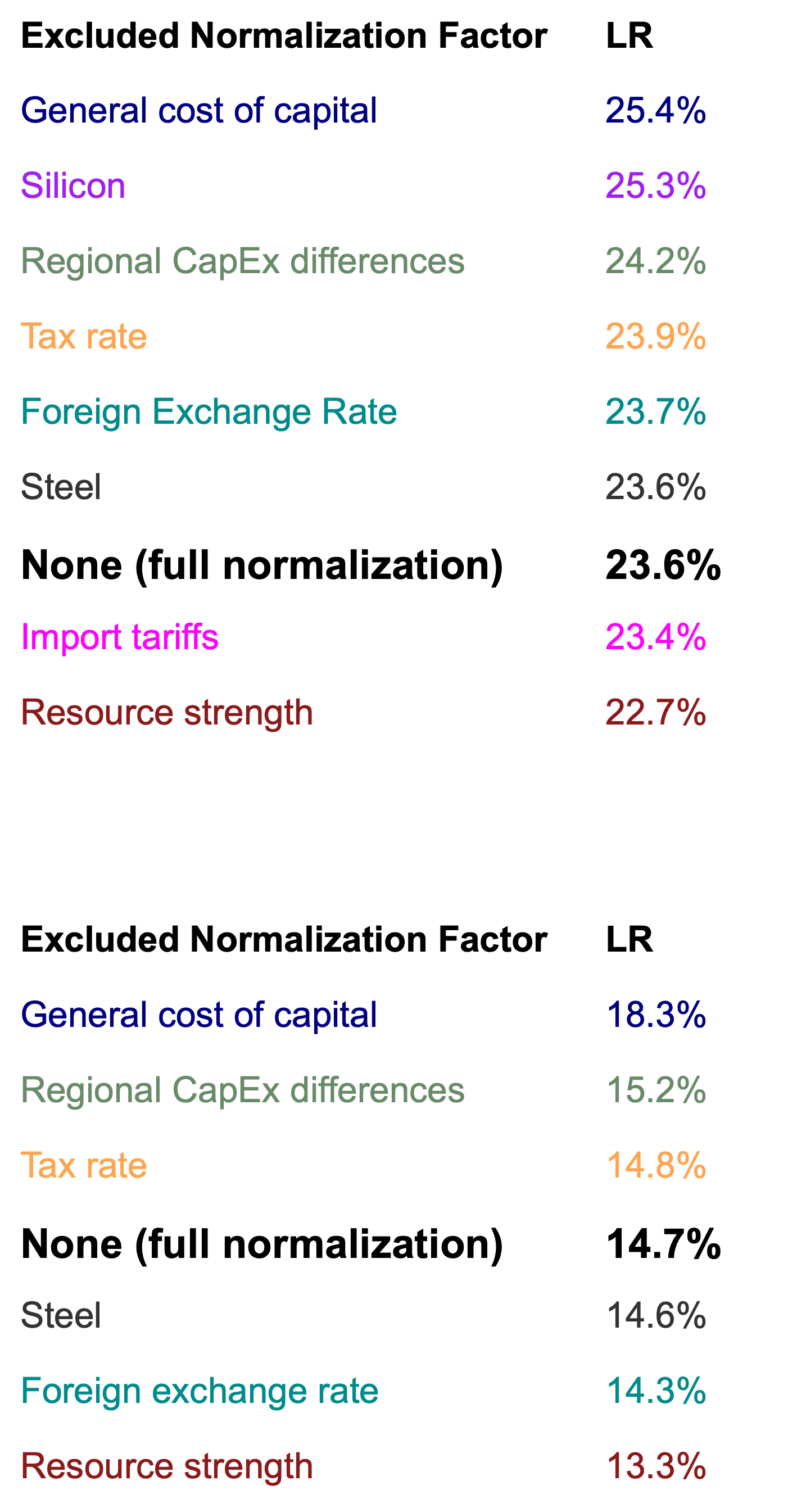

Supplement: Data S1. Data and Scripts, related to Figures 1–7, STAR Methods, and Document S1 — Data factor_delta_data: Data related to Figure 5 lcoe_learning_data: Annual datasets with inputs to all learning calculations, includes separate files for solar and wind (related to Figures 1–4, 6, and 7) project_level: Project-level LCOE estimates (related to Figure 1) SI Data: Additional data files related to Figure S1, S2, and S4; Tables S1–S4 and Tables S13–S16 Scripts figures_1_2_4_5: Scripts to generate Figures 1, 2, 4, and 5 forecasts: Script to generate LCOE-learning based forecasts (related to Figures 6 and 7) learning_curves: Scripts to run segmented regression models (related to Figure 3) prep: A base script that is run to load all data (this is run automatically within the other scripts) SI Scripts: Additional scripts related to Figures S1, S2, and S4; Tables S1–S4 and Tables S13–S16. [file mmc2.zip › Data and Scripts/Figures/SI/sensLeg.png]

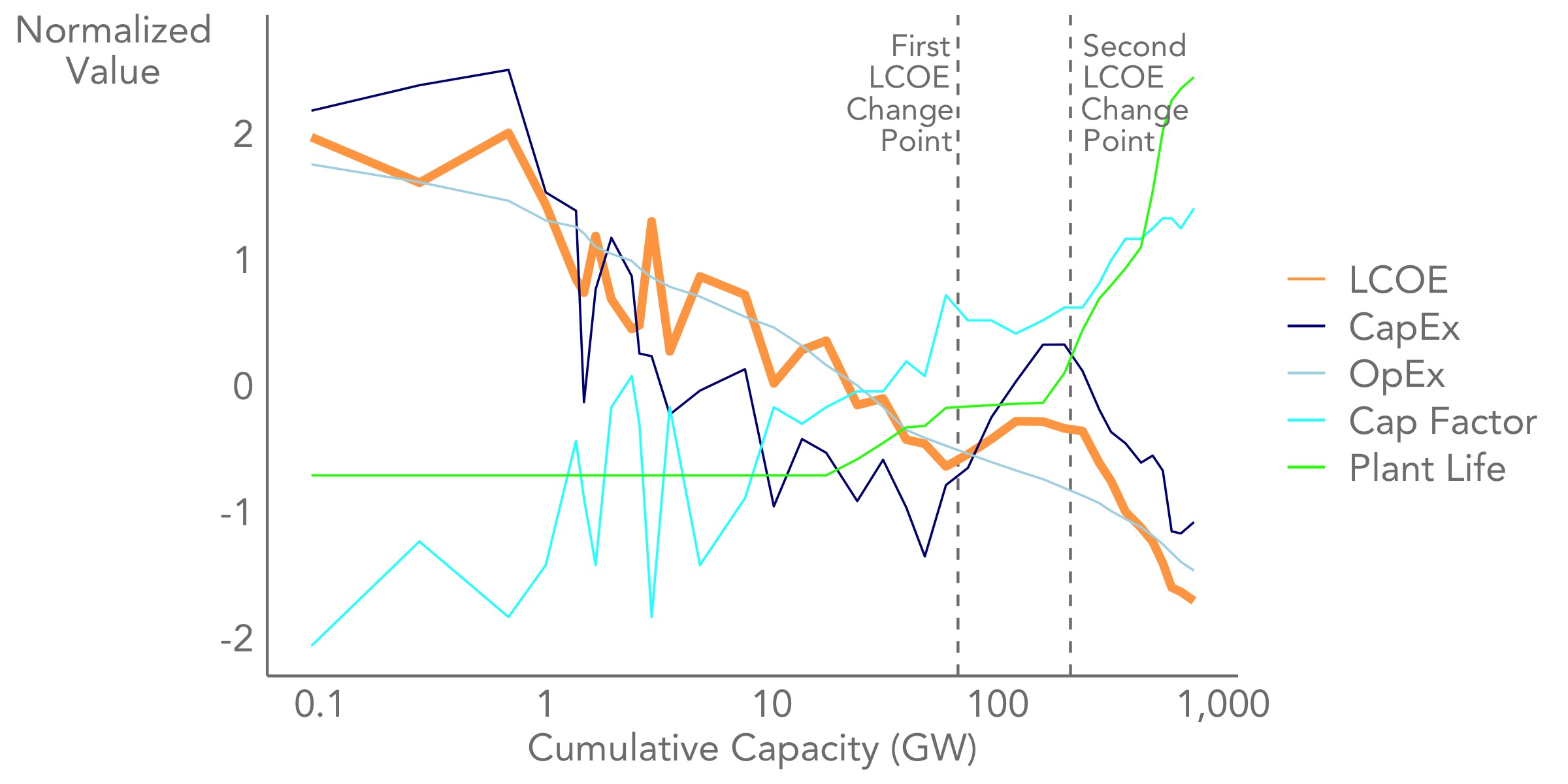

Supplement: Data S1. Data and Scripts, related to Figures 1–7, STAR Methods, and Document S1 — Data factor_delta_data: Data related to Figure 5 lcoe_learning_data: Annual datasets with inputs to all learning calculations, includes separate files for solar and wind (related to Figures 1–4, 6, and 7) project_level: Project-level LCOE estimates (related to Figure 1) SI Data: Additional data files related to Figure S1, S2, and S4; Tables S1–S4 and Tables S13–S16 Scripts figures_1_2_4_5: Scripts to generate Figures 1, 2, 4, and 5 forecasts: Script to generate LCOE-learning based forecasts (related to Figures 6 and 7) learning_curves: Scripts to run segmented regression models (related to Figure 3) prep: A base script that is run to load all data (this is run automatically within the other scripts) SI Scripts: Additional scripts related to Figures S1, S2, and S4; Tables S1–S4 and Tables S13–S16. [file mmc2.zip › Data and Scripts/Figures/SI/components_wind.jpg]

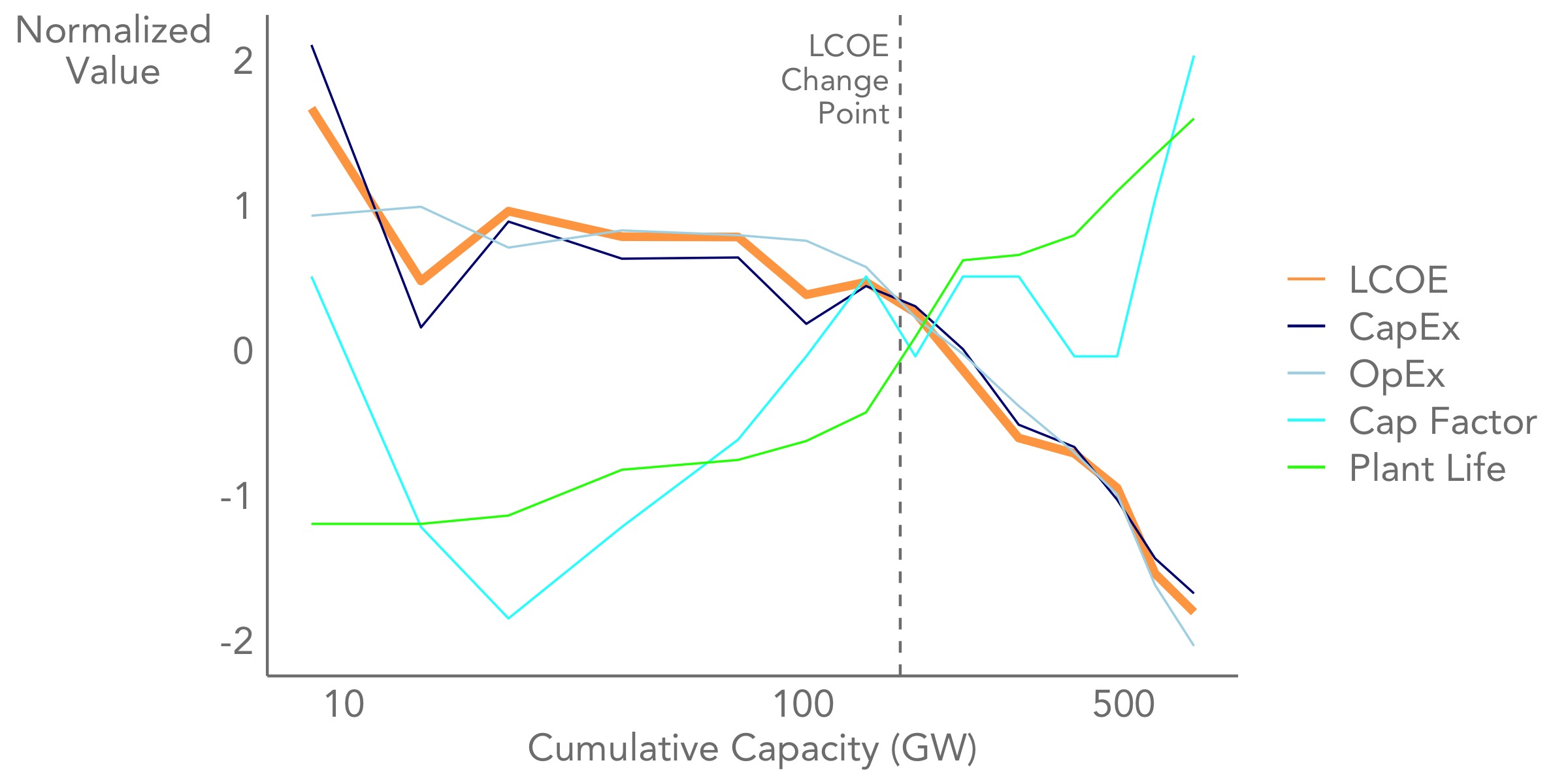

Supplement: Data S1. Data and Scripts, related to Figures 1–7, STAR Methods, and Document S1 — Data factor_delta_data: Data related to Figure 5 lcoe_learning_data: Annual datasets with inputs to all learning calculations, includes separate files for solar and wind (related to Figures 1–4, 6, and 7) project_level: Project-level LCOE estimates (related to Figure 1) SI Data: Additional data files related to Figure S1, S2, and S4; Tables S1–S4 and Tables S13–S16 Scripts figures_1_2_4_5: Scripts to generate Figures 1, 2, 4, and 5 forecasts: Script to generate LCOE-learning based forecasts (related to Figures 6 and 7) learning_curves: Scripts to run segmented regression models (related to Figure 3) prep: A base script that is run to load all data (this is run automatically within the other scripts) SI Scripts: Additional scripts related to Figures S1, S2, and S4; Tables S1–S4 and Tables S13–S16. [file mmc2.zip › Data and Scripts/Figures/SI/components_solar.jpg]
